# *G*-optimal designs for hierarchical linear models: an equivalence theorem and a nature-inspired meta-heuristic algorithm

**DOI:** 10.1007/s00500-021-06061-0

**Published:** 2021-08-07

**Authors:** Xin Liu, RongXian Yue, Zizhao Zhang, Weng Kee Wong

**Affiliations:** 1 College of Science, Donghua University, Shanghai, 201600 China; 2 Department of Mathematics, Shanghai Normal University, Shanghai, 200234 China; 3 Department of Biostatistics, University of California at Los Angeles, Los Angeles, CA 90095-1772 USA

**Keywords:** Approximate design, Locally *D*-optimal design, Poisson regression model, Random-effects model, Prediction

## Abstract

Hierarchical linear models are widely used in many research disciplines and estimation issues for such models are generally well addressed. Design issues are relatively much less discussed for hierarchical linear models but there is an increasing interest as these models grow in popularity. This paper discusses the *G*-optimality for predicting individual parameters in such models and establishes an equivalence theorem for confirming the *G*-optimality of an approximate design. Because the criterion is non-differentiable and requires solving multiple nested optimization problems, it is much harder to find and study *G*-optimal designs analytically. We propose a nature-inspired meta-heuristic algorithm called competitive swarm optimizer (CSO) to generate *G*-optimal designs for linear mixed models with different means and covariance structures. We further demonstrate that CSO is flexible and generally effective for finding the widely used locally *D*-optimal designs for nonlinear models with multiple interacting factors and some of the random effects are correlated. Our numerical results for a few examples suggest that *G* and *D*-optimal designs may be equivalent and we establish that *D* and *G*-optimal designs for hierarchical linear models are equivalent when the models have only a random intercept only. The challenging mathematical question of whether their equivalence applies more generally to other hierarchical models remains elusive.

## Introduction

Hierarchical models are widely used to analyze data in various disciplines, such as in psychology, medicine, manufacturing industry and education. Such models are especially appealing for analyzing longitudinal analysis because they allow for the presence of missing data, time-varying or invariant covariates, and subjects measured at different time points. In educational research, hierarchical models are commonly used to evaluate the effectiveness of teaching methods using data from students nested within classrooms and classrooms are nested within schools that use different teaching methods. The distinguishing feature of these models is that they account for both individual-level and population-level effects. In the literature across disciplines, hierarchical models are variously referred to as multilevel models, nested data models, mixed models, random coefficient, random-effects models or random parameter models. In our work, we make no distinction among them and refer to them as statistical models with parameters that vary at one or more levels.

When the experimental settings are under the control of the investigator, design issues arise and they must be carefully addressed for maximal precision in the statistical inference at a minimal cost. The basic questions to answer are given a statistical model defined on a compact design space ${\mathcal {X}}$, an optimality criterion and a fixed amount of resources to take *N* observations for the study, what are the optimal number of points, where these points are and how many replicates to take at each of these points. We denote these quantities by *k*, $x_1,\ldots ,x_k$ and $n_1,\ldots ,n_k$, respectively where $n_1+\cdots +n_k=N$. Optimal exact designs are challenging to find and study because there are no theoretical tools for finding them in general and in addition to depending on the design criterion and the model, the optimal exact design also depends on the value of *N*.

An alternative option is to formulate the design problem to find an optimal approximate design, where we determine *k*, $x_1,\ldots ,x_k$ as before, and now the optimal weights defined by $w_i=n_i/N\in [0,1]$ with $w_1+\cdots +w_k=1$. In practice, there are implemented by taking rounding each $Nw_j$ to the nearest integer $[Nw_j]$ and taking $[Nw_j]$ observations at $x_j,j=1\ldots ,k,$ subject to $[Nw_1]+\cdots +[Nw_k]=N$. Approximate designs were proposed in Kiefer (1959) and they are appealing because when the criterion is a concave functional, there is a theoretical tool called an equivalence theorem for confirming the optimality of an approximate design among all designs on the given design space ${\mathcal {X}}$. There are algorithms with proof of convergence for searching some types of optimal designs and there are also tools to assess the proximity of an approximate design from the optimum without knowing the latter. For these reasons, we focus on approximate designs in the rest of the paper.

A primary interest of our paper is to find *G*-optimal designs for hierarchical linear models, with *G* standing for global. These designs are best for estimating the overall response surface with minimal variance across the design space ${\mathcal {X}}$ and so protect against the worst-case scenario. This is unlike the much simpler situation where there is interest in finding an optimal design for estimating the predicted response at a single point. *G*-optimality is a minimax-type of design criterion, it is non-differentiable and requires at least solving two nested layers of optimization problems over different search spaces. Consequently, even for fixed-effects models, *G*-optimal designs are among the most difficult to study mathematically. Compounding the optimization problem is that current algorithms for searching optimal designs cannot find *G*-optimal designs effectively, let alone one with proof of convergence.

The main aims of this paper are to find *G*-optimal designs for hierarchical linear models, propose a nature-inspired meta-heuristic algorithm to find them and develop an equivalence theorem to confirm the *G*-optimality of an approximate design for hierarchical linear models. We demonstrate the flexibility and effectiveness of the algorithm for finding *G*-optimal designs when the mean response is modeled by fractional polynomials and *D*-optimal designs for hierarchical nonlinear models with multiple interacting factors with possibly correlated random effects. We also establish that *D* and *G*-optimal designs for hierarchical linear models are equivalent when the models have only a random intercept only and pose the challenging mathematical question of whether their equivalence applies more generally to other hierarchical models.

The rest of the paper is organized as follows. Section 2 provides the background of hierarchical linear models and recent literature on constructing *D*-optimal designs for such models. Section 3 establishes an equivalence theorem for *G*-optimality and Sect. 4 presents *G*-optimal designs for various types of hierarchical models for relatively simple models. To find *G*-optimal designs for more complicated models, Sect. 5 introduces a nature-inspired meta-heuristic algorithm called competitive swarm optimizer (CSO) to find *G*-optimal designs for more complicated hierarchical linear models, such as, when the mean function is a fractional polynomial and the random effects may be correlated. We further show CSO can find locally *D*-optimal designs for estimating model parameters in a Poisson hierarchical model with multiple factors defined on a user-specified design space ${\mathcal {X}}$ and some random effects are correlated. Section 6 concludes with a discussion of possible equivalence between *D* and *G*-optimal designs for linear mixed models.

## Preliminaries and model specification

This section gives the background, a brief literature review on constructing optimal designs for hierarchical linear models before we describe our statistical models.

### Equivalence theorem

Constructing optimal designs for a given model under a specified criterion is challenging if the model is complex, and more so if the criterion is complicated. An analytical approach for finding optimal designs is limiting and is possible only for simple models with a couple of parameters, see examples in design monographs, such as, Fedorov (1972) and Berger and Wong (2009). A common practice is to find an optimal design under a set of restrictive conditions and hope that the design remains optimal under a broader setup. For instance, one may find the best two-point optimal design for the simple logistic model. Is the same design still optimal among all three-point designs? Equivalence theorem provides an answer, and more generally, is able to confirm whether the design is optimal among all designs of interest.

Specifically, suppose the given design space ${\mathcal {X}}$ is compact and the optimality criterion is formulated as a concave (or convex) function of approximate designs or, equivalently, as a function of the normalized Fisher Information matrix ${\varvec{M}}(\xi )$. The elements of this matrix are the expectation of the negative of the second derivatives of the log-likelihood function with respect to the parameters. As an example, suppose we have a standard linear regression model with the mean response

$$\begin{aligned} E{\varvec{y}}~=~{\varvec{f}}^T(x){\varvec{\beta }}, \end{aligned}$$

where ${\varvec{y}}$ is a univariate function and ${\varvec{\beta }}$ is the $p\times 1$ vector of unknown parameters. The expectation is over the model errors assumed to have independent normal variates with zero means and constant variance. A direct calculation shows the Fisher Information matrix is proportional to

$$\begin{aligned} \int _{\mathcal {X}}{\varvec{f}}(x){\varvec{f}}(x)^T\xi (\mathrm{d}x). \end{aligned}$$

A typical design goal is find a design so that the estimated ${\varvec{ \beta }}$ has a minimal covariance matrix. The most common design criterion is *D*-optimality and a *D*-optimal design satisfies

$$\begin{aligned} \xi _D=\text {arg max}_{\xi \in \varXi }~\text {ln}~|{\varvec{ M}}(\xi )| \end{aligned}$$

over all approximate designs on ${\mathcal {X}}$. Here $|{\varvec{ M}}|$ is the determinant of ${\varvec{ M}}$ and $\varXi$ is the set of all approximate designs on ${\mathcal {X}}$. After the *D*-optimal design $\xi _D$ is found, the worth of another design $\xi$ in terms of *D*-optimality is measured by its D-efficiency defined by

$$\begin{aligned} \left\{ \frac{|{\varvec{ M}}(\xi )|}{|{\varvec{ M}}(\xi _D)|}\right\} ^{1/p}. \end{aligned}$$

Clearly, the above ratio is between 0 and 1 and if it is equal to one half, the interpretation is that the design $\xi$ has to be replicated twice to do as well as the *D*-optimal design $\xi _D$. If $\xi _D$ in the denominator is replaced by another design $\xi ^*$, the ratio is the *D*-efficiency of $\xi$ relative to $\xi ^*$. The efficiency or relative efficiency of a design under another criterion is similarly defined.

The function $ln~|{\varvec{ M}}|$ is concave on $\varXi$ and using a standard convex analysis argument (Fedorov 1972; Pazman 1986), it can be shown that the equivalence theorem for *D*-optimality is as follows: $\xi _D$ is *D*-optimal if and only if for all $x\in {\mathcal {X}}$,

1 $$\begin{aligned} {\varvec{f}}(x)^T{\varvec{ M}}(\xi _D)^{-1}{\varvec{f}}(x)-p\le 0. \end{aligned}$$

The function on the left-hand side of the inequality is frequently called the sensitivity function of the design. As an example, if ${\varvec{f}}(x)^T=(1,x,x^2)$, the design space is ${\mathcal {X}}=[1,3]$ and we want to estimate all the three parameters as accurately as possible. A direct calculation shows the *D*-optimal design $\xi _D$ takes equal proportion of observations at $x=1,x=2$ and $x=3$ and one can directly verify its sensitivity function satisfies (1).

Each concave functional has a directional derivative which is used to derive its own unique equivalence theorem; see details in design monographs, such as Fedorov (1972) and Berger and Wong (2005). When ${\mathcal {X}}$ is an interval or a two-dimensional space, the optimality of a design can be readily checked by plotting the sensitivity function on the left-hand side of the above inequality across the design space and visually ascertain whether the conditions in the equivalence theorem are satisfied. Equivalence theorems are frequently used to confirm the optimality of an approximate design, and more importantly, to ascertain the efficiency of any approximate design. An example is Wong and Cook (1993), where they proposed an algorithm to find *G*-optimal designs for fixed-effects linear models and assess the quality of the generated design using an equivalence theorem.

### Brief review of optimal designs

There is a lot of work on finding optimal designs for various models in different fields and most concerned finding *D*-optimal designs for linear models; see references in Berger and Wong (2005) and those cited below. For nonlinear models, the information matrix depends on the unknown parameters and nominal values are required to replace them in the information matrix before the criterion is optimized. Because the resulting designs depend on the nominal values, they are called locally *D*-optimal designs and are the simplest to construct for nonlinear models and commonly used in practice when a single best guess for the parameters is available. However, where there are conflicting opinions from experts or prior information from previous studies, locally optimal designs become problematic to implement.

Two common design approaches for such a scenario are to adopt a Bayesian paradigm or a minimax approach. The latter design strategy was used by Berger et al. (2000) where they searched for the best *D*-optimal design over a plausible set of values for the nominal values. The minimax *D*-optimal design from Berger et al. (2000) minimizes the maximum inefficiencies that arise from misspecified values in the plausible set; variations of the theme are possible; see Chen et al. (2018). An appeal of this approach is that practitioners are likely able to provide a set of plausible nominal values for the model parameters than having to provide a prior distribution to implement the Bayesian optimal design. A disadvantage of the minimax approach is that minimax optimal designs are both theoretically and numerically more challenging to find than Bayesian optimal designs.

Random-effects models have more complicated equivalence theorems and a limited number of them are available. For instance, Fedorov and Hackl (1972) derived an equivalence theorem to confirm the *D*-optimality of an approximate design for a random coefficient regression model and Entholzner et al. (2005) obtained optimal or efficient designs for mixed models. Schmelter (2007b) showed that the search for an optimal design for hierarchical linear models could be restricted to the class of group-wise identical individual designs and Schmelter (2007a) noted that optimal designs found in the class of single-group designs remain optimal in a larger class having more group designs. Entholzner et al. (2005), Schmelter (2007a) and Schmelter (2007b) investigated optimal designs under random intercept models, random slope models and random coefficient cubic regression models, respectively. Debusho and Haines (2008) and Debusho and Haines (2011) constructed *V*- and *D*-optimal population designs for linear and quadratic regression models with a random intercept term. More recently, Prus and Schwabe (2016b) constructed optimal designs for predicting individual parameters in hierarchical models and called them *D*-optimal designs. They also extended their work to find interpolation and extrapolation optimal designs for random coefficient regression models in Prus and Schwabe (2016a). Further, Prus (2019) proposed a design criterion called *G*-optimality for predicting the response surface in hierarchical models and noted technical difficulties in finding such an optimal design, analytically or numerically, for more complicated models.

### Statistical models

Throughout, we denote the *j*th response from the *i*th subject by $y_{ij}$. The hierarchical model is defined on a user-defined compact design space ${\mathcal {X}}$ and given by

2 $$\begin{aligned} \begin{aligned} y_{ij}&= {\varvec{f}}^T(x_{ij}){\varvec{\beta }}_{i} + \varepsilon _{ij}, \\ j&=1,\dots , m_i; \;i=1, \dots , n, \end{aligned} \end{aligned}$$

where the *j*th observation from individual *i* is taken at the experimental setting $x_{ij}\in {\mathcal {X}}$, *n* is the number of individuals, $m_i$ is the number of observations from individual *i*, ${{\varvec{f}}}= (f_1, \ldots , f_p)^T$ is the vector of known regression functions, and ${\varvec{\beta }}_i = (\beta _{i1}, \ldots , \beta _{ip})^T$ is the individual parameter vector specifying the individual response. The observational errors $\varepsilon _{ij}$ are assumed to be centred with zero mean and homoscedastic and uncorrelated with common variance var$(\varepsilon _{ij})=\sigma ^2$. We assume that E(${\varvec{\beta }}_i$)=${\varvec{\beta }}=(\beta _1, \ldots , \beta _p)^T$ and Cov(${\varvec{\beta }}_i$)=$\sigma ^2{\varvec{D}}$ ($i=1,\ldots ,n$), where ${\varvec{D}}$ is known, and all ${\varvec{\beta }}_i$’s are uncorrelated with all $\varepsilon _{ij}$’s. We note that the $p\times p$ matrix ${\varvec{D}}$ can be singular, which happens when some of the individual parameters are non-random.

In practice, experiments are usually conducted with identical regimes for all individuals, i.e., all individuals *i* have the same number $m_i=m$ of observations at the same values $x_{ij}=x_j$ of the experimental settings. Such designs are popular because they are simple to implement and analyze (Prus and Schwabe 2016b). In what is to follow, we assume such a setting.

Let ${\varvec{Y}}_i = ({\varvec{Y}}_{i1}, \ldots , {\varvec{Y}}_{im})^T$ be the vector of observations from individual *i*, let $\bar{{\varvec{Y}}} = \frac{1}{n}\sum _i^n {\varvec{Y}}_i$ be the average response across all individuals and let ${\varvec{F}} = ({{\varvec{f}}}(x_1), \ldots , {{\varvec{f}}}(x_m))^T$ be the individual $m\times p$ common design matrix for all individuals. Prus and Schwabe (2016b) showed that the best linear unbiased estimator $\hat{{\varvec{\beta }}}$ of the population parameter ${\varvec{\beta }}$ is

3 $$\begin{aligned} \hat{{\varvec{\beta }}}=({\varvec{F}}^T{\varvec{F}})^{-1}{\varvec{F}}^T\bar{{\varvec{Y}}}, \end{aligned}$$

and the best linear unbiased predictor $\hat{{\varvec{\beta }}}_i$ of the individual parameter ${\varvec{\beta }}_i$ is a weighted average of the individualized estimate $\hat{{\varvec{\beta }}}_{i;ind } = ({\varvec{F}}^T{\varvec{F}})^{-1}{\varvec{F}}^T{\varvec{Y}}_i$ based on the observations of subject *i* only and the estimator $\hat{{\varvec{\beta }}}$ for the population parameter. Specifically, we have

4 $$\begin{aligned} \begin{aligned} \hat{{\varvec{\beta }}}_i&= {\varvec{D}}\{({\varvec{F}}^T{\varvec{F}})^{-1} + {\varvec{D}})^{-1}\hat{{\varvec{\beta }}}_{i;ind } \\&\quad + ({\varvec{F}}^T{\varvec{F}})^{-1}(({\varvec{F}}^T{\varvec{F}})^{-1} + {\varvec{D}})^{-1}\hat{{\varvec{\beta }}}\}. \end{aligned} \end{aligned}$$

The quality of the prediction in (4) for ${\varvec{\theta }}=({\varvec{\beta }}^T_1, \ldots , {\varvec{\beta }}^T_n)^T$ may be measured by the mean squared error matrix of $\hat{{\varvec{\theta }}}=(\hat{{\varvec{\beta }}}^T_1, \ldots , \hat{{\varvec{\beta }}}^T_n)^T$:

5 $$\begin{aligned} \begin{array}{lll} MSE &{}=&{}Cov (\hat{{\varvec{\theta }}}-{\varvec{\theta }}) \\ &{}=&{} \sigma ^2\Big \{\frac{1}{n}{\varvec{J}}_n\otimes ({\varvec{F}}^T{\varvec{F}})^{-1} \\ &{}&{}+\, \Big ({\varvec{I}}_n-\frac{1}{n}{\varvec{J}}_n\Big )\otimes ({\varvec{D}}-{\varvec{D}}(({\varvec{F}}^T{\varvec{F}})^{-1}\\ &{}&{} + {\varvec{D}})^{-1}{\varvec{D}})\Big \}. \end{array} \end{aligned}$$

Here ${\varvec{I}}_n$ is the $n\times n$ identity matrix, ${\varvec{J}}_n$ is the $n\times n$ matrix with all entries equal to 1 and “$\otimes$” denotes the Kronecker product of matrices.

## An equivalence theorem for *G*-optimality

### *G*-optimality for predicting individual parameters

The *G*-optimality criterion was proposed for prediction of the individual parameters in Prus (2019) and a *G*-optimal design minimizes the maximal prediction mean squared error over the experimental region. The standardized information matrix of an approximate design $\xi$ for the above model without individual effects is

6 $$\begin{aligned} {{\varvec{M}}}(\xi )=\sum _{l=1}^k\frac{n_l}{m}{{\varvec{f}}}(x_l){{\varvec{f}}}^T({x_l})=\frac{1}{m}{\varvec{F}}^T{\varvec{F}}. \end{aligned}$$

The matrix ${{\varvec{M}}}(\xi )$ stands for the information obtained per observation and $m{{\varvec{M}}}(\xi )$ corresponds to the information contributed by the observations at the experimental settings per individual. In this paper, we are only concerned with approximate designs on ${\mathcal {X}}$ with non-singular information matrices and denote this set by $\varXi$. If $\xi \in \varXi$, its MSE-matrix in (5) is

$$\begin{aligned} \begin{aligned} MSE (\xi )&=\frac{\sigma ^2}{m}\Big \{\frac{1}{n}{\varvec{J}}_n\otimes {{\varvec{M}}}^{-1}(\xi )\\&\quad +\,\Big ({\varvec{I}}_n-\frac{1}{n}{\varvec{J}}_n\Big )\otimes ({\varvec{\varDelta }}-{\varvec{\varDelta }}({{\varvec{M}}}^{-1}(\xi )+ {\varvec{\varDelta }})^{-1}{\varvec{\varDelta }})\Big \}, \end{aligned} \end{aligned}$$

where ${\varvec{\varDelta }}=m{\varvec{D}}$. When ${\varvec{D}}$ is non-singular, this expression simplifies to

$$\begin{aligned} \begin{aligned} MSE ({\xi })&=\frac{\sigma ^2}{m}\Big \{\frac{1}{n}{\varvec{J}}_n\otimes {{\varvec{M}}}^{-1}(\xi )\\&\quad +\,\Big ({\varvec{I}}_n-\frac{1}{n}{\varvec{J}}_n\Big )\otimes ({{\varvec{M}}}(\xi )+ {\varvec{\varDelta }}^{-1})^{-1}\Big \}. \end{aligned} \end{aligned}$$

For predicting purposes, Prus (2019) used model (2) and proposed the *G*-criterion as the maximal sum of the expected squared differences of the predicted and real response across all individuals with respect to all possible observational settings:

7 $$\begin{aligned} \psi ^\mathrm{pred}_G(\xi )=\max _{x\in {\mathcal {X}}} \sum _{i=1}^nE \{({{\varvec{f}}}^T(x)(\hat{{\varvec{\beta }}}_i - {\varvec{\beta }}_i))^2\}. \end{aligned}$$

This criterion can be rewritten as

8 $$\begin{aligned} \psi ^\mathrm{pred}_G(\xi )=\max _{x\in {\mathcal {X}}}\phi (x,\xi ), \end{aligned}$$

where

9 $$\begin{aligned} \begin{aligned} \phi (x,\xi )&={{\varvec{f}}}^T(x){{\varvec{M}}}^{-1}(\xi ){{\varvec{f}}}(x) \\&\quad +\, (n-1){{\varvec{f}}}^T(x)({\varvec{\varDelta }}- {\varvec{\varDelta }}({{\varvec{M}}}^{-1}(\xi )\\&\quad +{\varvec{\varDelta }})^{-1}{\varvec{\varDelta }}){{\varvec{f}}}(x). \end{aligned} \end{aligned}$$

In the appendix, we show that the criterion is a convex function on $\varXi$ and a design that minimizes the *G*-criterion $\psi ^\mathrm{pred}_G(\xi )$ over all designs $\xi \in \varXi$ is *G*-optimal. The next two analytical results give a lower bound for the function $\psi ^\mathrm{pred}_G(\xi )$ for any design and an equivalence theorem to confirm the optimality of a design. The technical arguments are deferred to the appendix.

#### Theorem 1

For any design $\xi \in \varXi$, we have

10 $$\begin{aligned} \max _{x\in {\mathcal {X}}}\phi (x,\xi )\ge p + (n-1)\text {tr}\{({{\varvec{M}}}^{-1}(\xi ) + {\varvec{\varDelta }})^{-1}{\varvec{\varDelta }}\} \ge 0.\nonumber \\ \end{aligned}$$

An implication of the above theorem is that a design $\xi ^*$ is *G*-optimal for model (2) if it satisfies (10) with equality at the support points. In what is to follow, we establish below an equivalence theorem for *G*-optimality to characterize *G*-optimal designs.

#### Theorem 2

For model (2), let $\xi \in \varXi$, let ${\varvec{\varDelta }}=m{\varvec{D}}$, let $N(\xi ,{\varvec{\varDelta }})={\varvec{\varDelta }}-{\varvec{\varDelta }}({{\varvec{M}}}^{-1}(\xi )+{\varvec{\varDelta }})^{-1}{\varvec{\varDelta }}$, and let

$$\begin{aligned} \phi _G(x,\xi )= & {} {{\varvec{f}}}^T (x){{\varvec{M}}}^{-1} (\xi ){{\varvec{M}}}_{{\mathcal {A}}}(\mu ){{\varvec{M}}}^{-1} (\xi ){{\varvec{f}}}(x)\\&+\,(n-1){{\varvec{f}}}^T(x){{\varvec{N}}}(\xi ,{\varvec{\varDelta }}){{\varvec{M}}}_{{\mathcal {A}}}(\mu ){{\varvec{N}}}(\xi ,{\varvec{\varDelta }}){{\varvec{f}}}(x), \end{aligned}$$

where $\mu$ is a probability measure on ${\mathcal {A}}(\xi )$ defined by

$$\begin{aligned}{\mathcal {A}}(\xi )=\left\{ x\in {\mathcal {X}}\bigg |\phi (x,\xi )={\bar{\phi }}(\xi )=max_{z\in {\mathcal {X}}}~\phi (z,\xi ) \right\} \end{aligned}$$

with $${{\varvec{M}}}_{{\mathcal {A}}}(\mu )=\int _{{\mathcal {A}}(\xi )}{{\varvec{f}}}(x){{\varvec{f}}}^T(x)d \mu .$$

A design $\xi ^*$ on ${\mathcal {X}}$ is *G*-optimal if and only if there exists a probability measure $\mu ^*$ on ${\mathcal {A}}(\xi ^*)$ such that for all $x\in {\mathcal {X}}$

$$\begin{aligned}&\phi _G(x,\xi ^*) - tr \{{{\varvec{M}}}_{{\mathcal {A}}}(\mu ^*){{\varvec{M}}}^{-1}(\xi ^*) + (n - 1)\\&\quad {{\varvec{M}}}(\xi ^*){{\varvec{N}}}(\xi ^*,{\varvec{\varDelta }}){{\varvec{M}}}_{{\mathcal {A}}}(\mu ^*){{\varvec{N}}}(\xi ^*,{\varvec{\varDelta }})\}\le 0. \end{aligned}$$

Similar to (1), the function on the left hand side of the inequality is the sensitivity function of the design $\xi ^*$. The equivalence theorem is more complex than the one for *D*-optimality because the *G*-optimality criterion is not differentiable. The theorem has a form similar to those for confirming *G*-optimal designs for heteroscedastic linear models in Wong and Cook (1993).

In Theorem 2, the probability measure $\mu ^*$ exists only if the design under investigation is *G*-optimal, whereupon the inequality becomes an equality at all design points of the optimal design $\xi ^*$. This follows because if there is a support point *x* of $\xi ^*$ such that the inequality in Theorem 2 is strict, then integrating both sides with respect to $\xi ^*$, we have

11 $$\begin{aligned} \begin{aligned}&\int _{{\mathcal {X}}}\phi _G(x,\xi ^*)\mathrm{d}\xi ^*\\&\quad =\int _{{\mathcal {X}}}\text {tr}\left( {{\varvec{f}}}^T (x){{\varvec{M}}}^{-1} (\xi ^*){{\varvec{M}}}_{{\mathcal {A}}}(\mu ^*){{\varvec{M}}}^{-1} (\xi ^*){{\varvec{f}}}(x)\right. \\&\qquad \left. +\,(n-1){{\varvec{f}}}^T (x){{\varvec{N}}}(\xi ^*,{\varvec{\varDelta }}){{\varvec{M}}}_{{\mathcal {A}}}(\mu ^*){{\varvec{N}}}(\xi ^*,{\varvec{\varDelta }}){{\varvec{f}}}(x)\right) \mathrm{d}\xi ^*\\&\quad =\int _{{\mathcal {X}}}\text {tr}\left( {{\varvec{M}}}^{-1} (\xi ^*){{\varvec{M}}}_{{\mathcal {A}}}(\mu ^*){{\varvec{M}}}^{-1} (\xi ^*){{\varvec{f}}}(x){{\varvec{f}}}^T (x)\right. \\&\qquad \left. +\,(n-1){{\varvec{N}}}(\xi ^*,{\varvec{\varDelta }}){{\varvec{M}}}_{{\mathcal {A}}}(\mu ^*){{\varvec{N}}}(\xi ^*,{\varvec{\varDelta }}){{\varvec{f}}}(x){{\varvec{f}}}^T (x)\right) \mathrm{d}\xi ^*\\&\quad =\text {tr}\left( \left( {{\varvec{M}}}^{-1} (\xi ^*){{\varvec{M}}}_{{\mathcal {A}}}({}^*u){{\varvec{M}}}^{-1} (\xi ^*)\right. \right. \\&\qquad \left. \left. +\,(n-1){{\varvec{N}}}(\xi ^*,{\varvec{\varDelta }}){{\varvec{M}}}_{{\mathcal {A}}}(\mu ^*){{\varvec{N}}}(\xi ^*,{\varvec{\varDelta }})\right) \int _{{\mathcal {X}}}{{\varvec{f}}}(x){{\varvec{f}}}^T (x)\mathrm{d}\xi ^*\right) \\&\quad =\text {tr}\left( \left( {{\varvec{M}}}^{-1} (\xi ^*){{\varvec{M}}}_{{\mathcal {A}}}(\mu ^*){{\varvec{M}}}^{-1} (\xi ^*)\right. \right. \\&\qquad \left. \left. +\,(n-1){{\varvec{N}}}(\xi ^*,{\varvec{\varDelta }}){{\varvec{M}}}_{{\mathcal {A}}}(\mu ^*){{\varvec{N}}}(\xi ^*,{\varvec{\varDelta }})\right) {{\varvec{M}}}(\xi ^*)\right) \\&\quad =tr \left\{ {{\varvec{M}}}_{{\mathcal {A}}}(\mu ^*){{\varvec{M}}}^{-1}(\xi ^*)\right. \\&\quad \left. +\,(n - 1){{\varvec{M}}}(\xi ^*){{\varvec{N}}}(\xi ^*,{\varvec{\varDelta }}){{\varvec{M}}}_{{\mathcal {A}}}(\mu ^*){{\varvec{N}}}(\xi ^*,{\varvec{\varDelta }})\right\} , \end{aligned} \end{aligned}$$

implying $0 < 0$, which is impossible. It follows that all support points of $\xi ^*$ are roots of the sensitivity function. In the next section, we show how this information is used to generate the *G*-optimal design.

## *G*-optimal designs

We now provide examples of *G*-optimal designs for several types of linear mixed models and use Theorem 2 to confirm their optimality. Here and in the rest of the paper, to fix ideas, we assume that $n = 10, m = 5$ but other values for *n* and *m* can be similarly used. The first example is relatively simple and a formula for the *G*-optimal is available. When the model is slightly generalized to two additive factors with uncorrelated random factors and no intercept term, an analytical description for the *G*-optimal design for two-parameter models is no longer possible and numerical methods must be used. The implication is that *G*-optimal designs are difficult to find and they have to be found numerically. This motivates us to use competitive swarm optimizer (CSO) to find *G*-optimal designs and note that some of the results in this section are found by CSO.

### Examples

#### *G*-optimal design for the simple linear model with a random slope

Suppose we wish to find the *G*-optimal design for model (2) with $p = 2$ and ${{\varvec{f}}}(x) = (1, x)^T$ on the experimental region ${\mathcal {X}}= [0,1]$. The model is

12 $$\begin{aligned} y_{ij}= \beta _{1}+\beta _{i2}x_j+\varepsilon _{ij} \end{aligned}$$

and we assume that the slope parameter $\beta _{i2}$ is random with mean $\beta _2$ and the dispersion matrix is diagonal and equal to $\sigma ^2{\varvec{D}} = diag~(0, \mathrm{d}\sigma ^2)$.

Let $\delta =md$ and let $\xi ^*_w$ be the two-point design supported at 0 and 1 with weight at 1 equal to $w^*$. A direct application of Theorem 2 shows that the *G*-optimal design for model (12) has

13 $$\begin{aligned} w^*=\frac{\sqrt{\delta ^2n^2 + 4\delta + 4} + n\delta - 2}{2 \delta (n+1)} \end{aligned}$$

and for the design $\xi _w^*$, (9) becomes

14 $$\begin{aligned} \phi (x,\xi _w^*)= & {} \left( \frac{1}{w^*- w^{*2} } + \frac{\delta (n - 1)}{\delta w^* + 1}\right) x^2 + \frac{2}{w^* - 1}x \nonumber \\&- \frac{1}{w^* - 1}. \end{aligned}$$

This maximum of this function is attained at $x=0$ and $x=1$ and so ${\mathcal {A}}(\xi _w^*)=\left\{ 0,1\right\} .$ Let $\mu ^*$ be the two-point probability measure defined on ${\mathcal {A}}(\xi _w^*)$ supported at 0 and 1 and its weight at 1 is

$$\begin{aligned} \begin{aligned} w_{\mu }&=(\delta ^2w^{*4} + 2\delta w^{*3} + w^{*2})/[ (n+1)\delta ^2w^{*4} \\&\quad + (4\delta - 2n\delta ^2)w^{*3} + (2 - 4\delta \\&\quad +n \delta ^2)w^{*2} + 2(\delta -1)w^* + 1]. \end{aligned} \end{aligned}$$

The sensitivity function of the design $\xi _w^*$ in terms of $w_{\mu }$ and $w^*$ is

15 $$\begin{aligned} \phi _G(x,\xi _w^*)= & {} \left( \frac{w^{*2} - 2w^*w_{\mu }+w_{\mu }}{w^{*2}(w^* - 1)^2} + \frac{\delta ^2w_{\mu }(n - 1)}{(\delta w^* + 1)^2}\right) x^2 \nonumber \\&\,+ \frac{2(w_{\mu } - 1)}{(w^* - 1)^2}x + \frac{1-w_{\mu }}{(w^* - 1)^2} \end{aligned}$$

with a maximum value of ${(1-w_{\mu })}/{(w^* - 1)^2}$ at $x=0$ and $x=1$. This value can be shown to equal to

$$\begin{aligned} \begin{aligned}&tr \left\{ {{\varvec{M}}}_{{\mathcal {A}}}(\mu ^*){{\varvec{M}}}^{-1}(\xi _w^*) \right. \\&\quad \left. +\,(n - 1){{\varvec{M}}}(\xi _w^*){{\varvec{N}}}(\xi _w^*,{\varvec{\varDelta }}){{\varvec{M}}}_{{\mathcal {A}}}(\mu ^*){{\varvec{N}}}(\xi _w^*,{\varvec{\varDelta }})\right\} \end{aligned} \end{aligned}$$

and so by Theorem 2, the design $\xi _w^*$ is *G*-optimal.

#### *G*-optimal designs for a two additive factor model without an intercept

Regression models with no intercept are quite common and they either arise naturally or from constraints imposed on the variables, see for example, Huang et al. (1995). For such a model with two additive random factors, we have

16 $$\begin{aligned} y_{ij}= \beta _{i1}x_{1j}+\beta _{i2}x_{2j}+\varepsilon _{ij}, \end{aligned}$$

where $(x_{1j}, x_{2j})\in {\mathcal {X}}=[0, 1]^2$. The dispersion matrix of ${\varvec{\beta }}_i=(\beta _{i1},\beta _{i2})^T$ is diagonal and equal to $\sigma ^2{\varvec{D}} = diag~(d_1\sigma ^2, d_2\sigma ^2)$. To find *G*-optimal designs, first consider designs of the form

$$\begin{aligned} \xi _w=\left\{ \begin{array}{ccc} (1,0) &{}\quad (0,1)&{}\quad (1,1) \\ w_1 &{}\quad w_2 &{}\quad w_3 \end{array}\right\} ~\text {with}~\quad w_1+w_2+w_3=1, \end{aligned}$$

and let $\mu _w$ be the associated probability measure on $A(\xi _w)$ defined by

$$\begin{aligned}&\mu _w=\left\{ \begin{array}{ccc} (1,0) &{}\quad (0,1)&{}\quad (1,1) \\ w_{\mu 1} &{}\quad w_{\mu 2} &{}\quad w_{\mu 3} \end{array}\right\} ~\text {with}~ \\&w_{\mu 1} + w_{\mu 2} + w_{\mu 3}=1. \end{aligned}$$

The table below shows the optimal weights $$\xi ^*_w$$ and the weights of the measure $$\mu ^*_w$$ for model (16) for selected values of $$d_1$$ and $$ d_2.$$ These designs have been verified to be numerically *G*-optimal by Theorem 2.

**Table:** 

$d_1$	$d_2$	$w_1^*$	$w_2^*$	$w_3^*$	$w_{\mu 1}^*$	$w_{\mu 2}^*$	$w_{\mu 3}^*$
1	1	0.2788	0.2788	0.4423	0.3272	0.3272	0.3456
	5	0.2312	0.3674	0.4014	0.3268	0.3355	0.3377
	10	0.2251	0.3789	0.3960	0.3269	0.3361	0.3371
5	1	0.3674	0.2312	0.4014	0.3355	0.3268	0.3377
	5	0.3215	0.3215	0.3569	0.3331	0.3331	0.3337
	10	0.3156	0.3333	0.3511	0.3331	0.3333	0.3336
10	1	0.3789	0.2251	0.3960	0.3361	0.3269	0.3371
	5	0.3333	0.3156	0.3511	0.3333	0.3331	0.3336
	10	0.3274	0.3274	0.3452	0.3333	0.3333	0.3334

#### *G*-optimal designs for quadratic and fractional polynomial mixed models

We show here that even for a relatively simple mixed model, an analytic description of the *G*-optimal design can be problematic. We consider two linear mixed models with independent errors $\epsilon _{ij}$ having zero means and common variance. These two models are

17 $$\begin{aligned} \begin{aligned} y_{ij}&= \beta _{i0} + \beta _{i1}x_j + \beta _{i2}x_j^2 + \epsilon _{ij},\\ j&= 1,\ldots , m,\; i = 1, \ldots , n, \end{aligned} \end{aligned}$$

and

18 $$\begin{aligned} \begin{aligned} y_{ij}&= \beta _{i0} + \beta _{i1}x_j^{1/2} + \beta _{i2}x_j + \beta _{i3}x_{j}^2 + \epsilon _{ij}, \\ j&= 1,\ldots , m,\; i = 1, \ldots , n. \end{aligned} \end{aligned}$$

Model (17) is quadratic and model (18) is an example of a fractional polynomial, which is increasingly used in the biomedical sciences to model a univariate continuous response. Fractional polynomial models were proposed by Royston and Altman (1994) who showed that they are more effective for modeling a continuous outcome than using polynomials (Royston et al. 1999; Royston and Sauerbrei 2008). We recall a fractional polynomial (FP) is given by $\phi _m(x;\mathbf {\alpha },{\mathbf {p}})=\alpha _0+\sum _{j=1}^t \alpha _j H_j(x)$, where $\alpha _j$ are the real-valued coefficients and $H_j(x)$ are defined sequentially, $H_1(x)=x^{(p_1)}$,

$$\begin{aligned} H_j(x)=\left\{ \begin{array}{ll} x^{(p_j)}, &{}\quad \text{ if } p_j\ne p_{j-1},\\ H_{j-1}(x) \ln [x], &{}\quad \text{ if } p_j=p_{j-1}, \end{array} \right. \text{ for } j=2,\ldots ,t. \end{aligned}$$

The powers are given by the Box-Tidwell transformation with $x^{(p_j)}=x^{p_j}$ if $p_j\ne 0$, otherwise $x^{(0)}=\ln [x]$ and for practical applications, ‘powers’ in a FP are selected from the set ${\mathcal {P}}=\{-2,-1,-0.5,0,0.5,1,2, \ldots ,\max (3,t)\}$ (Royston and Altman 1994). Many software statistical packages now provide an option for fitting FP models, suggesting that FP models are gaining recognition as a modelling tool in statistics. Interestingly, optimal designs for FP models have never been reported in the literature.

There are no closed-form descriptions for the optimal designs for these two relatively simple models with random effects. A practical way is to find them using an algorithm. There are many traditional algorithms for generating many types of optimal designs and our experience is that many of them do not work well in such a setting where the criterion is not differentiable and the optimization problem has two levels. To this end, we use a meta-heuristic algorithm called competitive swarm optimizer to find the optimal designs.

## Competitive swarm optimizer

An analytical approach is generally unable to determine a *G*-optimal design and we need an effective algorithm to generate a *G*-optimal design, or search for a design with sufficiently high *G*-efficiency for practical applications. The last few *G*-optimal designs in Sect. 4 were found by an algorithm that we now describe.

Nature-inspired meta-heuristic algorithms are commonly used in engineering and computer science to tackle hard-to-solve optimization problems (Yang 2010). Examples are particle swarm optimization (PSO), differential evolutionary (DE), cuckoo search (CS) and imperialist competitive algorithm (ICA). Their main appeal is that they are general purpose optimization tools, they tend to be assumptions-free, easy to implement and use and frequently able to find high-quality solutions quickly. Their meteoric rise in popularity is well documented in Whitacre (2011a, 2011b) with reasons. Recently, Chen et al. (2018), Phoa et al. (2016), Kim and Wong (2018), Masoudi et al. (2019), and Storn and Price (1997) used meta-heuristic algorithms to tackle different types of optimal design problems. For example, Chen et al. (2018) applied a version of PSO to find standardized maximin optimal designs for several enzyme-kinetic inhibition models by solving multilevel nested optimization problems over different types of search spaces, and Kim and Wong (2018) likewise applied PSO and solved an adaptive clinical trial design problem by solving a complex discrete optimization problem by determining the optimal choice of ten integers with multiple constraints.

Evolutionary algorithms are continuously evolving and nature-inspired meta-heuristic algorithms are a major component of evolutionary algorithms. Competitive swarm optimizer (CSO) is popular because many simulation results in the literature show that it either outperforms or is competitive with several state-of-the-art evolutionary algorithms. This conclusion was arrived at after comparing CSO’s performance with several state-of-the-art evolutionary algorithms using a variety of benchmark functions with dimensions up to 5000 (Cheng and Jin 2014; Zhou et al. 2016; Sun et al. 2018; Mohapatra et al. 2017; Zhang et al. 2016). They showed that CSO was frequently not only the winner but also required significantly less run time. CSO has also been successfully applied to solve many different types of complex optimization problems; see, for example, Gu et al. (2018), Kumarappan and Arulraj (2016) and Xiong and Shi (2018).

CSO was initially proposed by Cheng and Jin (2014) to tackle the premature convergence issue met by many evolutionary algorithms. CSO first generates a swarm of *n* particles at positions ${\mathbf {x}}_1, \ldots , {\mathbf {x}}_n$ with random velocities ${\mathbf {v}}_1, \ldots , {\mathbf {v}}_n$ in $\varvec{\varOmega }$. In each iteration, we randomly divide them into $\left\lfloor \frac{n}{2} \right\rfloor$ pairs and compare their objective function values. If we have a minimization problem, at iteration *t*, we compare each pair of particles ${\mathbf {x}}^t_i$ and ${\mathbf {x}}^t_j$ and identify ${\mathbf {x}}^t_i$ as the winner and ${\mathbf {x}}^t_j$ as the loser if the objective function has a smaller value at ${\mathbf {x}}^t_i$ than at ${\mathbf {x}}^t_j$. Winner retains status quo and the loser learns from the winner. The two defining equations for CSO are

$$\begin{aligned}&{\mathbf {v}}^{t+1}_{j} = {\mathbf {R}}_1 \otimes {\mathbf {v}}^t_{j} + {\mathbf {R}}_2 \otimes ({\mathbf {x}}^t_{i} - {\mathbf {x}}^t_{j}) +\gamma {\mathbf {R}}_3 \otimes (\bar{{\mathbf {x}}}^t - {\mathbf {x}}^t_{j}) \\&\quad \text {and}~{\mathbf {x}}^{t+1}_{j} = {\mathbf {x}}^t_{j} + {\mathbf {v}}^{t+1}_{j}, \end{aligned}$$

where ${\mathbf {R}}_1, {\mathbf {R}}_2, {\mathbf {R}}_3$ are all random vectors whose elements are drawn from *U*(0, 1); operation $\otimes$ also represents element-wise multiplication; vector $\bar{{\mathbf {x}}}^t$ is simply the swarm center at iteration *t*; social factor $\gamma$ controls the influence of the neighboring particles to the loser and a large value is helpful for enhancing the swarm diversity (but possibly impacts convergence rate). This process iterates until some stopping criteria are met. Algorithm 1 displays a pseudo code of CSO.
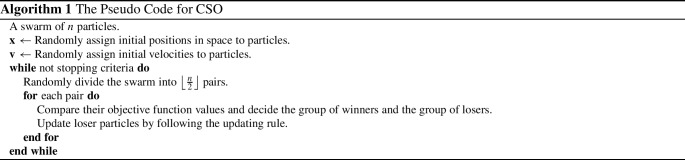


The tuning parameters we used in the CSO algorithm for finding the optimal designs are similar to those suggested by Cheng and Jin (2014). For example, because the optimization problems we have dimensions fewer than 100, we set $\gamma = 0$ and used 128 particles in the search. This is because the examples to follow have about 100 variables or fewer to optimize. The maximum number of iterations for each search was set to be 350. For our examples, the algorithm typically converges in 200 or fewer iterations or less than 1 second of CPU time. The hardware we used is a Windows PC with 3.20GHz Intel i7-8700 CPU, 32GB DDR4 2666MHz memory and 512G SSD storage. Here convergence means that successive values of the objective function do not differ by less than $10^{-6}$ in absolute value. On average, we observe that each search usually converged in 200 or fewer iterations.

### Application of CSO to find *G*-optimal designs

To search for a *G*-optimal design, we set up a two-layer optimization structure for min-max the objective function. The inner optimization step is a low-dimensional maximization problem represented in formula (9) and the outer loop is to minimize formula (8), which, in our cases, is a multi-dimensional function with less than 100, or more specifically, around 10–20 variables to optimize. Accordingly, we set $\gamma =0$, 32 particles, 128 iterations for the inner optimization and $\gamma = 0$, 128 particles and 200 iterations for the outer optimization task. During the search, the target matrix may sometimes become singular or close to being singular and affect the floating-point calculation accuracy. We found adding a diagonal matrix with very small positive diagonal elements to the original matrix can make inverting an ill-conditioned matrix more stable. In our work, we set ${\varvec{ M}}^*= {\varvec{M}} + 0.0000001 \times {\mathbf {I}}.$

After finding a design $\xi ^*$, we determine its answering set ${\mathcal {A}}(\xi ^*)$ and the probability measure $\mu ^*$ that meet the conditions in the equivalence theorem. To efficiently find all *x*’s that maximize $\phi (x, \xi ^*)$, we suggest to split the design space into two or more subspaces and then run CSO on each subspace to search for all *x*’s that maximize $\phi (x, \xi ^*)$. For instance, if the design space ${\mathcal {X}} = [0, 1]$, we may split the search space into [0, 0.5] and [0.5, 1]. There are no firm rules for the number of subspaces and our suggestion is to first try with two subspaces, then aggregate all such points to obtain ${\mathcal {A}}(\xi ^*)$. We then sequentially increase the number of subspaces and stop the process when splitting the design space into more subspace does not enlarge the size of ${\mathcal {A}}(\xi ^*)$.

To find the probability measure $\mu ^*$ that meets the conditions in the equivalence theorem, we proceed iteratively as follows. For each generated design $\xi$ from the algorithm, we first determine elements $a_1,a_2,\ldots ,a_k$ in $A(\xi )$ and find a candidate $\mu$ for $\mu ^*$ among probability measures supported at the *k* points. There are 2*k* variables in the pairs $(a_i,w_i)$, where $w_i$ is the weight of $\mu$ at $a_i, i=1, \ldots , k$. Since the support points of $\mu ^*$ must be roots of the sensitivity function when $\xi$ is optimal, one may apply CSO to minimize the following function with respect to the variable weights $w_i$:

19 $$\begin{aligned} \begin{aligned}&\min _{\mu } \;[\sup _{x}\phi _G(x,\xi ) -tr\{{\varvec{M}}_{{\mathcal {A}}}(\mu ){\varvec{M}}^{-1}(\xi )\\&\quad +\, (n-1){\varvec{N}}(\xi , \varvec{\varDelta }){\mathbf {M}}_{{\mathcal {A}}}(\mu ){\varvec{N}}(\xi , \varvec{\varDelta })\}]^2 \\&\quad +\, \sum ^k_{i=1}w_i[\phi _G(x_i, \xi ) -tr\{{\mathbf {M}}_{{\mathcal {A}}}(\mu ){\varvec{M}}^{-1}(\xi ) \\&\quad + (n-1){\varvec{N}}(\xi , \varvec{\varDelta }){\mathbf {M}}_{{\mathcal {A}}}(\mu ){\varvec{N}}(\xi , \varvec{\varDelta })\}]^2. \end{aligned} \end{aligned}$$

We note that in (19), for each design $\xi$, we have to first find *x* that maximizes $\phi _G(x,\xi )$. This can be done by calling CSO again to tackle this optimization problem. However, after a lot of experiments, we find that only minimizing the second term in (19) frequently suffices to determine $\mu$, i.e. we suggest given a design, first find $\mu$ by simply minimizing

20 $$\begin{aligned} \begin{aligned}&\min _{\mu } \sum ^k_{i=1}w_i[\phi _G(x_i, \xi ) -tr\{{\mathbf {M}}_{{\mathcal {A}}}(\mu ){\varvec{M}}^{-1}(\xi ) \\&\quad +\, (n-1){\varvec{N}}(\xi , \varvec{\varDelta }){\mathbf {M}}_{{\mathcal {A}}}(\mu ){\varvec{N}}(\xi , \varvec{\varDelta })\}]^2 \end{aligned} \end{aligned}$$

before solving the more complicated problem (19) to determine $\mu ^*$. We ran CSO with $\phi =0.05$, 64 particles for 1200 iterations. We stop the algorithm if and when the objective value attains a small user pre-specified value of, say, $10^{-5}$.

The CSO-generated designs for the next few examples were found by optimizing (20) and we confirm their *G*-optimality via the equivalence theorem. On average, the CPU time required to solve (20) was about 5 seconds or less for our examples and we expect a longer time is required to solve (19). This suggests that if we want to find G-optimal designs for more complicated problems, an efficient strategy to find *G*-optimal designs is to first solve the simpler optimization problem in (20) before solving (19).

The next few tables list CSO-generated designs for various linear mixed models with different assumptions on the covariance structure of the random effects and their *G*-optimality criterion values. We display the sensitivity plot of each CSO-generated design across the design space and it confirms the *G*-optimality of the CSO-generated design. All parameters for the design problems, such as the design space, the elements in the covariance matrix ${\varvec{D}}$, *n* and *m* are chosen randomly for illustrative purposes. The error variance $\sigma ^2$ is a nuisance parameter and does not affect the optimization process, so we set $\sigma ^2=1$ in all examples.

**Table 1:** CSO-generated design and its features for model (17) for specific choices of the covariance matrix of the uncorrelated random effects, design space and (*n*, *m*)

Model	$y_{ij} = \beta _{i0} + \beta _{i1}x_j + \beta _{i2}x_j^2 + \epsilon _{ij}$
${\varvec{D}}$	*diag*(0.2, 0.2, 0.3)
Design space	[0, 2]
(*n*, *m*)	(10, 5)
*G*-optimal design	$$\begin{pmatrix} 0.000 &{} 0.966 &{} 2.000 \\ 0.146 &{} 0.140 &{} 0.714 \end{pmatrix}$$
*G*-criterion value	13.480
$\mu ^*$ on $A(\xi )$	$$\begin{pmatrix} 0.000 &{} 0.948 &{} 2.000 \\ 0.231 &{} 0.175 &{} 0.595 \end{pmatrix}$$
Plot of $\phi _G(x,\xi )$	Figure 1

**Table 2:** CSO-generated design and its features for model (17) for specific choices of the covariance matrix of the correlated random effects, design space and (*n*, *m*)

Model	$y_{ij} = \beta _{i0} + \beta _{i1}x_j + \beta _{i2}x_j^2 + \epsilon _{ij}$
${\varvec{D}}$	$$\begin{pmatrix} 0.80 &{} 0.30 &{} 0.10 \\ 0.30 &{} 0.50 &{} 0.08 \\ 0.10 &{} 0.08 &{} 0.40 \end{pmatrix}$$
Design space	[0, 3]
(*n*, *m*)	(11, 4)
*G*-optimal design	$$\begin{pmatrix} 0.000 &{} 1.274 &{} 3.000 \\ 0.165 &{} 0.270 &{} 0.565 \end{pmatrix}$$
*G*-criterion value	19.000
$\mu ^*$ on $A(\xi )$	$$\begin{pmatrix} 0.000 &{} 1.243 &{} 3.000 \\ 0.207 &{} 0.338 &{} 0.455 \end{pmatrix}$$
Plot of $\phi _G(x,\xi )$	Figure 2

**Table 3:** CSO-generated design and its features for model (18) for specific choices of the covariance matrix of the uncorrelated random effects, design space and (*n*, *m*)

Model	$y_{ij} = \beta _{i0} + \beta _{i1}x_j^{1/2} + \beta _{i2}x_j + \beta _{i3}x_j^2+ \epsilon _{ij}$
${\varvec{D}}$	*diag*(0.3, 0.5, 0.8, 0.2)
Design space	[1, 3]
(*n*, *m*)	(10, 5)
*G*-optimal design	$$\begin{pmatrix} 1.000 &{} 1.440 &{} 2.342 &{} 3.000 \\ 0.186 &{} 0.190 &{} 0.120 &{} 0.504 \end{pmatrix}$$
*G*-criterion value	17.939
$\mu ^*$ on $A(\xi )$	$$\begin{pmatrix} 1.000 &{} 1.431 &{} 2.338 &{} 3.000 \\ 0.233 &{} 0.224 &{} 0.100 &{} 0.443 \end{pmatrix}$$
Plot of $\phi _G(x,\xi )$	Figure 3

**Table 4:** CSO-generated design and its features for model (18) for specific choices of the covariance matrix of the correlated random effects, design space and (*n*, *m*)

Model	$y_{ij} = \beta _{i0} + \beta _{i1}x_j^{1/2} + \beta _{i2}x_j + \beta _{i3}x_j^2 + \epsilon _{ij}$
${\varvec{D}}$	$$\begin{pmatrix} 0.80 &{} 0.30 &{} 0.10 &{} 0.05 \\ 0.30 &{} 0.50 &{} 0.08 &{} 0.04 \\ 0.10 &{} 0.08 &{} 0.40 &{} 0.02 \\ 0.05 &{} 0.04 &{} 0.02 &{} 0.30 \end{pmatrix}$$
Design space	[1, 3]
(*n*, *m*)	(8, 4)
*G*-optimal design	$$\begin{pmatrix} 1.000 &{} 1.419 &{} 2.372 &{} 3.000 \\ 0.230 &{} 0.186 &{} 0.122 &{} 0.462 \end{pmatrix}$$
*G*-criterion value	15.546
$\mu ^*$ on $A(\xi )$	$$\begin{pmatrix} 1.000 &{} 1.410 &{} 2.360 &{} 3.000 \\ 0.274 &{} 0.198 &{} 0.106 &{} 0.422 \end{pmatrix}$$
Plot of $\phi _G(x,\xi )$	Figure 4

**Fig. 1:**
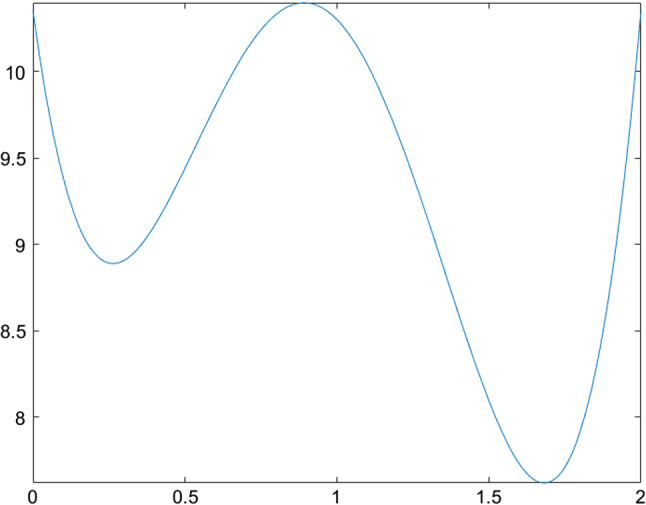
Sensitivity function of the CSO-generated for model (17) with uncorrelated random effects in Table 1

**Fig. 2:**
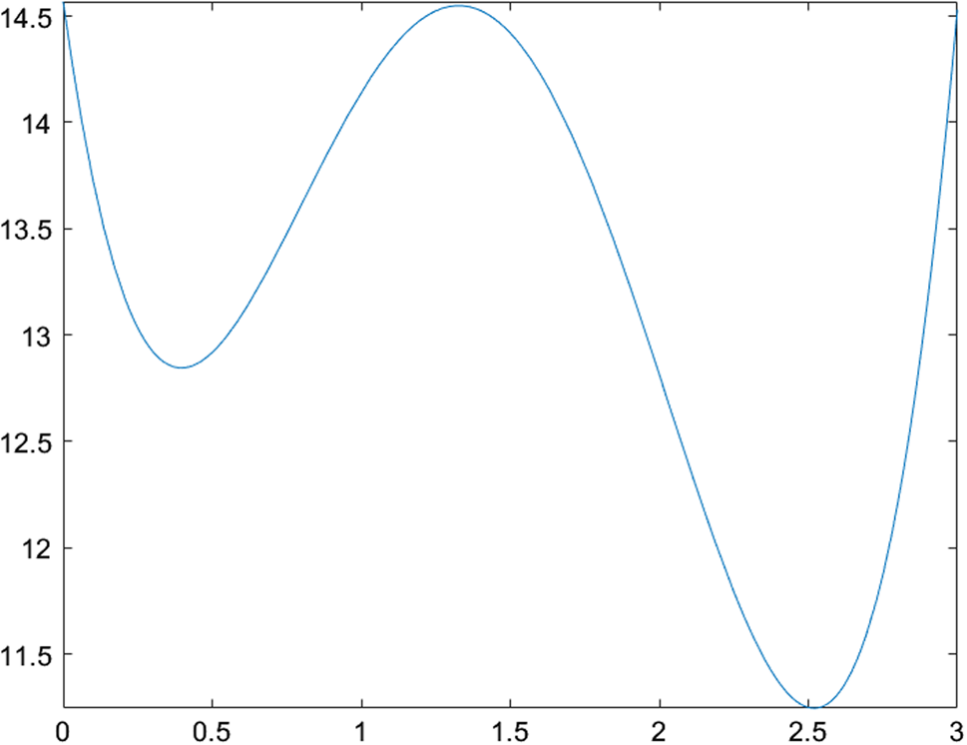
Sensitivity function of the CSO-generated for model (17) with correlated random effects in Table 2

**Fig. 3:**
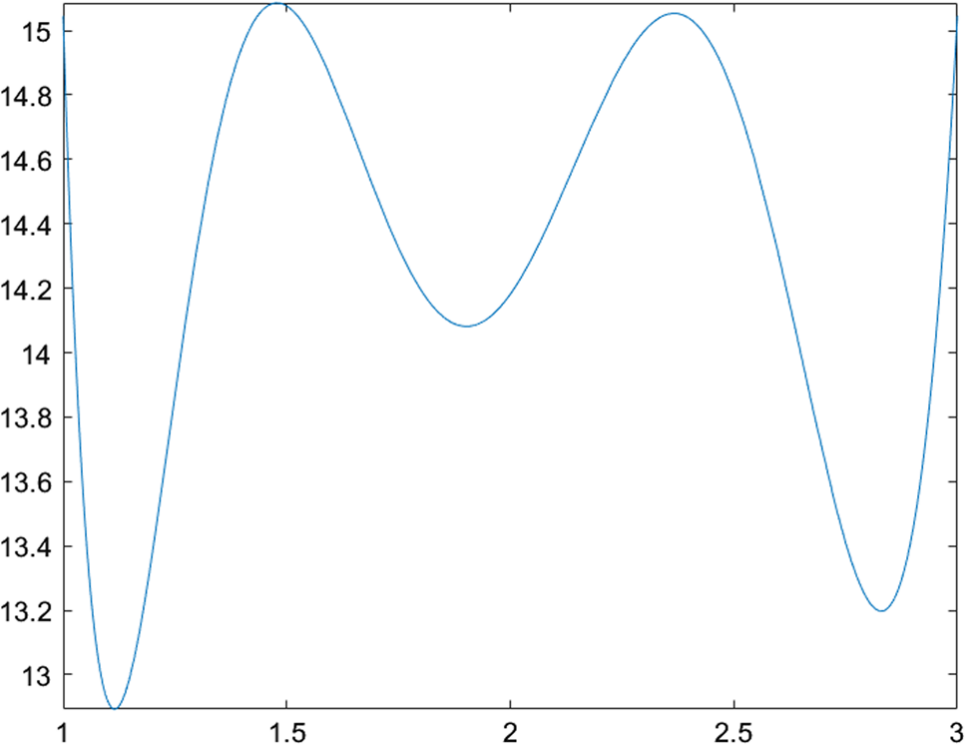
Sensitivity function of the CSO-generated design for model (18) with uncorrelated random effects in Table 3

**Fig. 4:**
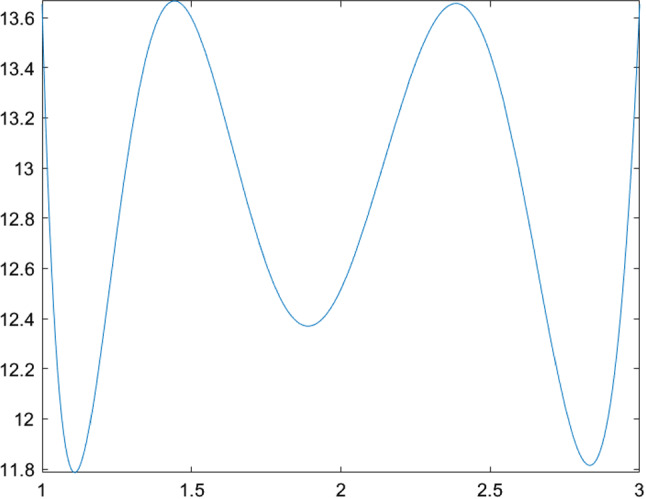
Sensitivity function of the CSO-generated design for model (18) with correlated random effects in Table 4

### CSO-generated locally *D*-optimal designs for Poisson models with mixed factors

This subsection demonstrates the utility and flexibility of CSO to find other types of optimal designs, such as locally *D*-optimal designs for estimating parameters in a Poisson regression model with possibly interacting factors and some factors are random.

Poisson models are commonly used to study count data in a regression setting even though they have restrictive assumptions, such as, requiring the mean and variance of the response to be equal. Negative binomial regression models extend Poisson models when the variance is larger or smaller than the mean response. These models are frequently used in clinical trials when the primary outcome is a count variable, such as the number of falls by an elderly patient, or number of CT scans received in the last three months or modeling the numbers of insurance claims and results. Walters (2007a, 2007b) used a negative binomial regression model to account for the number of aggressive incident reports in the following 12 months after subjects were put in an institutional correction center. Like all meta-heuristic algorithms, they can be modified to search for an optimum more effectively for specific problems. An enhanced version of CSO algorithm is available to find various types of optimal designs for the negative binomial regression models (Zhang et al. 2020).

Locally *D*-optimal designs for two-factor mixed Poisson models with an intercept term have been reported for two models, one with and the other without an interaction term. By using the quasi-information matrix defined in Niaparast and Schwabe (2013) and assuming that the random effects are uncorrelated, i.e., the covariance matrix is diagonal, Naderi et al. (2018) provided theoretical details for finding minimally supported *D*-optimal designs, including an equivalence theorem to confirm the *D*-optimality of an approximate design. We recall that minimally supported designs have the number of support points equal to the number of parameters in the mean function and so cannot be used to perform a lack of fit test to check model adequacy. Thus their approach can be restrictive in practice and it is also not clear whether their numerical procedure works for finding *D*-optimal designs when the model has more interacting factors with random effects or under another design criterion.

**Table 5:** A locally *D*-optimal design for a mixed Poisson model with an interaction term and uncorrelated random effects

Model	$Y_{ij} \sim P(\lambda _{ij}), \lambda _{ij} = \exp (\beta _{0i} + \beta _{1i}x_{ij,1} +\beta _{2i}x_{ij, 2} + \beta _{03}x_{ij,1}x_{ij,2})$
Covariance matrix ${\varvec{D}}$	*diag*(0.3, 0.2, 0.5, 0.6)
Coefficient mean $\varvec{\beta }$	$[-0.5, 0.2, -0.3, 0.4]$
Design space	$[-0.5, 1.7] \times [-1.0, 0.6]$
*D*-optimal design	$$\begin{pmatrix} -0.500 &{} -0.500 &{} 1.218 &{} 1.700 &{} 1.700 \\ -1.000 &{} 0.600 &{} -1.000 &{} -0.578 &{} 0.600 \\ 0.263 &{} 0.382 &{} 0.052 &{} 0.144 &{} 0.159 \end{pmatrix}$$
$\log |M(\xi )|$	− 2.660

**Table 6:** A locally *D*-optimal design for a mixed Poisson model with an interaction term and correlated random effects

Model	$Y_{ij} \sim P(\lambda _{ij}), \lambda _{ij} = \exp (\beta _{0i} + \beta _{1i}x_{ij,1} +\beta _{2i}x_{ij, 2} + \beta _{03}x_{ij,1}x_{ij,2})$
Covariance matrix ${\varvec{D}}$	$$\begin{pmatrix} 0.30 &{} 0.02 &{} 0.10 &{} 0.00 \\ 0.02 &{} 1.10 &{} 0.60 &{} 0.00 \\ 0.10 &{} 0.60 &{} 1.20 &{} 0.00 \\ 0.00 &{} 0.00 &{} 0.00 &{} 0.00 \end{pmatrix}$$
Coefficient mean $\varvec{\beta }$	$[-0.9, 1.0, 1.2, -1.5]$
Design space	$[-0.8, 1.4] \times [-1.3, 0.5]$
*D*-optimal design	$$\begin{pmatrix} -0.800 &{} 0.528 &{} 0.701 &{} 1.179 &{} 1.400 &{} 1.400 \\ 0.500 &{} -1.300 &{} 0.500 &{} -0.280 &{} -1.300 &{} 0.500 \\ 0.365 &{} 0.320 &{} 0.135 &{} 0.095 &{} 0.075 &{} 0.010 \end{pmatrix}$$
$\log |M(\xi )|$	− 4.211

**Fig. 5:**
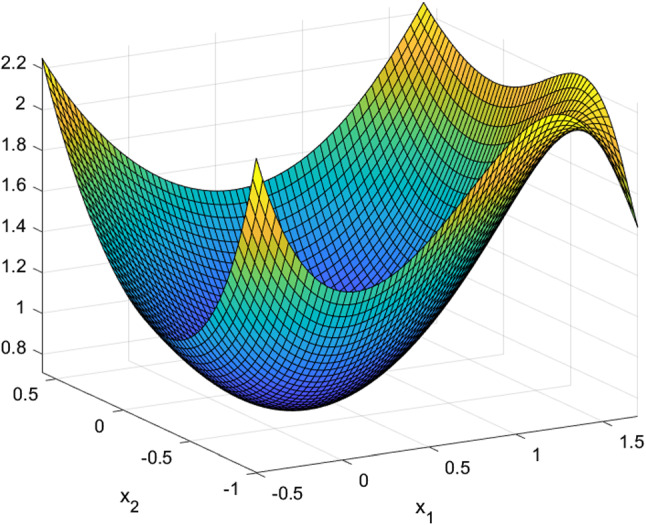
The sensitivity function of the CSO-generated design under the *D*-optimality criterion for the two-factor Poisson model with an interaction term and uncorrelated random effects shown in Table 5

**Fig. 6:**
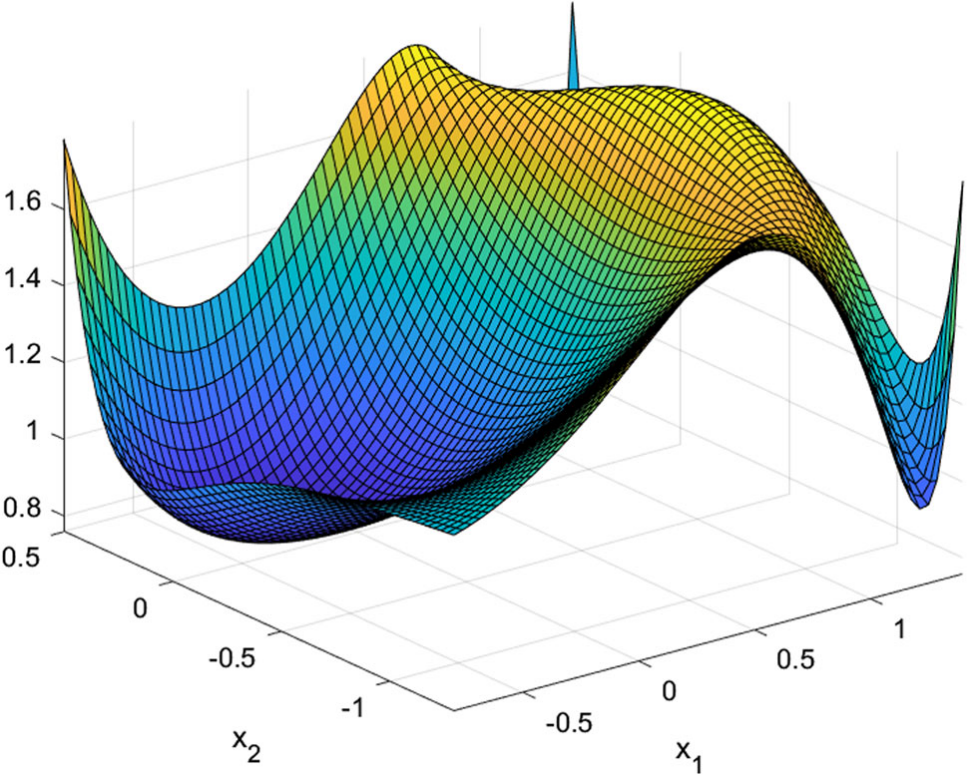
The sensitivity function of the CSO-generated design under the *D*-optimality criterion for a two-factor Poisson model with an interaction term and correlated random effects shown in Table 6

We applied CSO and found the same locally *D*-optimal designs in Tables 1, 2 and 3 of Naderi et al. (2018) for different models with various numbers of uncorrelated random coefficients. The next two sets of results in Tables 5 and 6 show that CSO-generated designs for a model with two interacting factors when the random effects are uncorrelated or correlated. The accompanying plots in Figs. 5 and 6 display their sensitivity plots and they confirm the CSO-generated designs are locally *D*-optimal. Interestingly, the results show different design spaces can produce *D*-optimal designs that are not minimally supported.

$$\begin{aligned}&\xi ^4= \left( \begin{array}{cccccccccccc} 0.056 &{}\quad -0.250 &{}\quad 0.474 &{}\quad 0.723 &{}\quad 0.369 &{}\quad -0.313 &{}\quad -0.322 &{}\quad 0.362 &{}\quad -0.220 &{}\quad 0.003 &{}\quad 0.321 &{}\quad -0.279 \\ 0.025 &{}\quad 0.726 &{}\quad 0.893 &{}\quad -0.658 &{}\quad -0.504 &{}\quad 0.533 &{}\quad -0.491 &{}\quad 0.501 &{}\quad 0.042 &{}\quad -0.003 &{}\quad 0.422 &{}\quad 0.847 \\ -1.000 &{}\quad 0.379 &{}\quad -0.305 &{}\quad -0.263 &{}\quad -0.182 &{}\quad -0.214 &{}\quad -0.298 &{}\quad -0.014 &{}\quad -1.000 &{}\quad -1.000 &{}\quad -0.195 &{}\quad -0.455 \\ 1.000 &{}\quad 0.789 &{}\quad 0.229 &{}\quad 0.176 &{}\quad -1.000 &{}\quad -1.000 &{}\quad -1.000 &{}\quad 1.000 &{}\quad 1.000 &{}\quad -1.000 &{}\quad -1.000 &{}\quad 0.407 \\ 0.261 &{}\quad 0.049 &{}\quad 0.013 &{}\quad 0.047 &{}\quad 0.048 &{}\quad 0.079 &{}\quad 0.096 &{}\quad 0.048 &{}\quad 0.106 &{}\quad 0.138 &{}\quad 0.096 &{}\quad 0.019 \\ \end{array}\right) \\&\xi ^{5} = \left( \begin{array}{cccccccccccc} -0.021 &{}\quad 0.510 &{}\quad -0.021 &{}\quad 0.519 &{}\quad -0.021 &{}\quad 0.238 &{}\quad -1.000 &{}\quad -1.000 &{}\quad -0.910 &{}\quad 0.510 &{}\quad -0.141 &{}\quad 0.020 \\ 1.000 &{}\quad 1.000 &{}\quad 1.000 &{}\quad -0.514 &{}\quad 1.000 &{}\quad -0.428 &{}\quad 0.625 &{}\quad 0.690 &{}\quad -0.176 &{}\quad 1.000 &{}\quad 0.534 &{}\quad 0.375 \\ -1.000 &{}\quad -0.299 &{}\quad -1.000 &{}\quad 0.341 &{}\quad -1.000 &{}\quad -0.546 &{}\quad 0.333 &{}\quad -0.485 &{}\quad -0.410 &{}\quad -0.299 &{}\quad -0.566 &{}\quad 0.360 \\ -1.000 &{}\quad -1.000 &{}\quad 1.000 &{}\quad -1.000 &{}\quad -1.000 &{}\quad -1.000 &{}\quad -1.000 &{}\quad -1.000 &{}\quad -1.000 &{}\quad -1.000 &{}\quad -1.000 &{}\quad -1.000 \\ 0.589 &{}\quad -1.000 &{}\quad -1.000 &{}\quad -1.000 &{}\quad -1.000 &{}\quad -1.000 &{}\quad -1.000 &{}\quad -1.000 &{}\quad -1.000 &{}\quad 0.389 &{}\quad -1.000 &{}\quad -1.000 \\ 0.224 &{}\quad 0.058 &{}\quad 0.208 &{}\quad 0.022 &{}\quad 0.091 &{}\quad 0.016 &{}\quad 0.063 &{}\quad 0.016 &{}\quad 0.062 &{}\quad 0.208 &{}\quad 0.009 &{}\quad 0.024 \\ \end{array}\right) . \end{aligned}$$

The usefulness of the CSO algorithm can also be seen when we applied it find *D*-optimal designs for mixed models when some random coefficients are correlated and able to find *D*-optimal designs that may not be minimally supported. We demonstrate using two Poisson models,one with four and the other with five mixed factors and both have some interaction terms. This is helpful because the bulk of theoretical optimal designs in the literature for nonlinear models have only a couple of additive factors and numerical results for models with interaction terms are also very limited.

For the model with four factors, we assume that

$$\begin{aligned} \begin{aligned} Y_{ij}&\sim P(\lambda _{ij}), \\ \lambda _{ij}&= \exp (\beta _{0i} + \beta _{1i}x_{ij,1} +\beta _{2i}x_{ij,2} +\beta _{3i}x_{ij,3} \\&\quad +\, \beta _{4i}x_{ij,4} +\beta _{5i}x_{ij,1}x_{ij, 2} + \beta _{6i}x_{ij, 1}x_{ij, 4} \\&\quad + \beta _{7i}x_{ij, 2}x_{ij, 4}), \end{aligned} \end{aligned}$$

the covariance matrix is ${\varvec{D}} = diag(0.5, 2.2, 1, 1, 0, 0, 1.3, 0.7)$ and the coefficients in the linear predictor function is $\varvec{\beta } = (1, 2, 3, -3, -1, -2, 1, 3)$. The 12-point CSO-generated design $\xi ^4$ is shown below and criterion value is $-4.300$. Here and elsewhere, the last row in the design for a multi-factor model shows the mass of the design point above it.

The second example concerns a Poisson regression model with five factors ($x_1,\ldots , x_5$) and three interaction terms ($x_1x_2, x_1x_3, x_3x_5$) defined on $[-1, 1]^5$. As an illustrative example, suppose the covariance matrix is

$$\begin{aligned} {\varvec{D}}= & {} Blockdiagonal({\varvec{D}}_1, {\varvec{D}}_2),\\ {\varvec{D}}_1= & {} \begin{pmatrix} 1.3 &{}\quad 0.6 &{}\quad 0.1 \\ 0.6 &{}\quad 1.0 &{}\quad 0.4 \\ 0.1 &{}\quad 0.4 &{}\quad 1.2 \\ \end{pmatrix},\\ {\varvec{D}}_2= & {} diag(1.0, 0.0, 0.0, 0.3, 0.5, 0.0) \end{aligned}$$

and the nominal coefficients in the linear predictor function $\varvec{\beta } = (1.0, 2.0, 3.0, -3.0, -1.0, -2.0, 0.2, 0.5, -0.5)$. The criterion value of the 12-point CSO-generated is $-13.640$ and the design $\xi ^{5}$ is shown below. This is a more complicated model and as expected, CSO, like other meta-heuristic algorithms, can also encounter problems when the optimization problem becomes more complex. Convergence and numerical stability issues can arise and repeated reruns of the algorithm with different tuning parameters and swarm size did not produce an optimal design, which is the case here.

A common strategy to try to overcome the above problem is to hybridize the algorithm with another algorithm, such that the hybridized version performs better than either of the algorithms. Another option is to use the equivalence theorem to derive an efficiency lower bound for the generated approximate design without knowing the optimum (Pazman 1986). For *D*-efficiency, the lower bound depends on the maximum value of the sensitivity function of the design. Since CSO is a stochastic algorithm and depends on the initial design and choice of tuning parameters, we ran the algorithm multiple times and reported the minimum of the *D*-efficiency of the generated design. For this example, the CSO generated design is $\xi ^{5}$ and its computed minimum *D*-efficiency is $88\%$, which may suffice in practice.

For models with three or more factors, like in the last two examples, the CSO-generated designs appear to be *D*-optimal or highly *D*-efficient. Unlike previous examples, the sensitivity functions are now high dimensional and so it is difficult to plot them and visually appreciate their features. One can discretize the design space using a fine grid evaluate the values of their sensitivity function at every grid point but this can be time-consuming. Another way is to apply CSO to optimize the multi-dimensional sensitivity function and confirm all peaks occur at the design points of the CSO-generated design. We chose the latter option and have verified that the CSO-generated design $\xi ^4$ is numerically optimal and $\xi ^{5}$ is not.

### Are *D* and *G*-optimal designs equivalent for hierarchical linear models?

It is well known that for regression models with homoscedastic errors, the *D* and *G*-optimal approximate designs are equivalent (Kiefer and Wolfowitz 1960). The two optimality criteria look different and have different purposes and so it is an intriguing result. Does the equivalence of the two types of designs apply when we have linear mixed regression models?

Let ${\varvec{J}}_n$ be the *n*-dimensional square matrix whose elements are all ones, let $\varvec{\varDelta } = m{\varvec{D}}$, and let $\otimes$ denote the Kronecker product. *D*-optimal design for model (2) minimizes the log $|\hbox {MSE}(\xi )|$ where

$$\begin{aligned} \begin{aligned} \text {MSE}(\xi )&= \frac{\sigma ^2}{m}\Big \{\frac{1}{n}{\varvec{J}}_n\otimes {\varvec{M}}^{-1}(\xi ) \\&\quad + \Big ({\varvec{I}}_n - \frac{1}{n}{\varvec{J}}_n\Big )\otimes (\varvec{\varDelta } - \varvec{\varDelta }({\varvec{M}}^{-1}(\xi )+\varvec{\varDelta })^{-1}\varvec{\varDelta })\Big \}. \end{aligned} \end{aligned}$$

Tables 7 and 8 display CSO-generated designs for the *D* and *G*-optimality criteria on different settings for the mixed quadratic model with random components having various covariance matrices. Tables 9 and 10 show corresponding results for a fractional polynomial model with random components having various covariance matrices. The numerical results suggest that *D* and *G*-optimal designs for these models are almost equivalent since their *D* and *G*-efficiencies relative to the other are all very close to 1. Additional numerical results not shown here for space consideration, for other models we have investigated with more polynomial or fractional polynomial terms and different types of covariance structures also show that the two types of optimal designs have relative efficiencies very close to 1.

Our numerical results in the tables suggest that the celebrated theorem of Kiefer and Wolfowitz may also apply to hierarchical linear models, but a general proof is elusive at this time. However, we are able to show the equivalence of the two types of designs holds for hierarchical linear models when they contain only a random intercept. To see this, we recall *D*-optimality discussed in Prus and Schwabe (2016b) for the hierarchical linear model (2). Assuming that the dispersion matrix ${{\varvec{D}}}$ has rank *q*, the *D*-criterion for prediction is the logarithm of the product of the $(n-1)q+p$ largest eigenvalues of the MSE-matrix:

$$\begin{aligned} \psi ^\mathrm{pred}_D(\xi )=\ln [\det \{{{\varvec{M}}}^{-1}(\xi ) \}]+(n-1)\ln \Big \{ \prod \limits _{l=1}^q \lambda _l(\xi ,{\varvec{\varDelta }}) \Big \}, \end{aligned}$$

where $\lambda _1(\xi ,{\varvec{\varDelta }}),\ldots ,\lambda _q(\xi ,{\varvec{\varDelta }})$ are the *q* largest eigenvalues of ${{\varvec{N}}}(\xi ,{\varvec{\varDelta }})={\varvec{\varDelta }}- {\varvec{\varDelta }}({\varvec{{{\varvec{M}}}}}^{-1}(\xi )+{\varvec{\varDelta }})^{-1}{\varvec{\varDelta }}$. Corollary 6 of Prus and Schwabe (2016b) used this definition and gave an equivalence theorem for *D*-optimality, and showed that the *D*-optimal design in the fixed-effects model is also *D*-optimal for prediction in the random intercept model.

**Table 7:** *D* and *G*-optimal designs for a quadratic mixed model with uncorrelated random effects

Model	$y_{ij} = \beta _{i0} + \beta _{i1}x_j + \beta _{i2}x_j^2 + \epsilon _{ij}$
${\varvec{D}}$	*diag*(0.2, 0.2, 0.3)
Design space	[0, 2]
(*n*, *m*)	(10, 5)
*G*-optimal design	$$\begin{pmatrix} 0.000 &{} 0.966 &{} 2.000 \\ 0.146 &{} 0.140 &{} 0.714 \end{pmatrix}$$
*G*-criterion value	13.480
*D*-efficiency	99%
*D*-optimal design	$$\begin{pmatrix} 0.000 &{} 0.946 &{} 2.000 \\ 0.157 &{} 0.140 &{} 0.703 \end{pmatrix}$$
*D*-criterion value	− 4.666
*G*-efficiency	99%

**Table 8:** *D* and *G*-optimal designs for a quadratic mixed model with correlated random effects

Model	$y_{ij} = \beta _{i0} + \beta _{i1}x_j + \beta _{i2}x_j^2 + \epsilon _{ij}$
${\varvec{D}}$	$$\begin{pmatrix} 0.80 &{} 0.30 &{} 0.10 \\ 0.30 &{} 0.50 &{} 0.08 \\ 0.10 &{} 0.08 &{} 0.40 \end{pmatrix}$$
Design space	[0, 3]
(*n*, *m*)	(11, 4)
*G*-optimal design	$$\begin{pmatrix} 0.000 &{} 1.274 &{} 3.000 \\ 0.165 &{} 0.270 &{} 0.565 \end{pmatrix}$$
*G*-criterion value	19.000
*D*-efficiency	99%
*D*-optimal design	$$\begin{pmatrix} 0.000 &{} 1.239 &{} 3.000 \\ 0.163 &{} 0.272 &{} 0.565 \end{pmatrix}$$
*D*-criterion value	− 8.798
*G*-efficiency	99%

**Table 9:** *D* and *G*-optimal designs for a fractional polynomial mixed model with uncorrelated random effects

Model	$y_{ij} = \beta _{i0} + \beta _{i1}x_j^{1/2} + \beta _{i2}x_j + \beta _{i3}x_j^2+ \epsilon _{ij}$
${\varvec{D}}$	*diag*(0.3, 0.5, 0.8, 0.2)
Design space	[1, 3]
(*n*, *m*)	(10, 5)
*G*-optimal design	$$\begin{pmatrix} 1.000 &{} 1.440 &{} 2.342 &{} 3.000 \\ 0.186 &{} 0.190 &{} 0.120 &{} 0.504 \end{pmatrix}$$
*G*-criterion value	17.939
*D*-efficiency	99%
*D*-optimal design	$$\begin{pmatrix} 1.000 &{} 1.412 &{} 2.361 &{} 3.000 \\ 0.230 &{} 0.186 &{} 0.122 &{} 0.462 \end{pmatrix}$$
*D*-criterion value	− 2.116
*G*-efficiency	99%

**Table 10:** *D* and *G*-optimal designs for a fractional polynomial mixed model with correlated random effects

Model	$y_{ij} = \beta _{i0} + \beta _{i1}x_j^{1/2} + \beta _{i2}x_j + \beta _{i3}x_j^2+ \epsilon _{ij}$
${\varvec{D}}$	$$\begin{pmatrix} 0.800 &{} 0.300 &{} 0.100 &{} 0.050\\ 0.300 &{} 0.500 &{} 0.080 &{} 0.040\\ 0.100 &{} 0.080 &{} 0.400 &{} 0.020 \\ 0.050 &{} 0.040 &{} 0.020 &{} 0.300\end{pmatrix}$$
Design space	[1, 4]
(*n*, *m*)	(10, 5)
*G*-optimal design	$$\begin{pmatrix} 1.000 &{} 1.602 &{} 2.914 &{} 4.000 \\ 0.206 &{} 0.218 &{} 0.109 &{} 0.467 \end{pmatrix}$$
*G*-criterion value	20.271
*D*-efficiency	99%
*D*-optimal design	$$\begin{pmatrix} 1.000 &{} 1.599 &{} 2.884 &{} 4.000 \\ 0.205 &{} 0.217 &{} 0.110 &{} 0.468 \end{pmatrix}$$
*D*-criterion value	− 89.985
*G*-efficiency	99%

We next consider using the *G*-optimal design for prediction in a hierarchical linear model with a random intercept. Assume $f_1(x)\equiv 1$ in model (2). The dispersion matrix ${{\varvec{D}}}$ can be written as ${{\varvec{D}}}=d{{\varvec{e}}}_1{{\varvec{e}}}_1^T$, where ${{\varvec{e}}}_1=(1, 0, \ldots , 0)^T$ denotes the first unit vector in ${\mathbb {R}}^p$. For an approximate design $\xi \in \varXi$, the MSE-matrix for prediction in this random-intercepts model is

$$\begin{aligned} MSE (\xi )= & {} \frac{\sigma ^2}{m}\Big \{\frac{1}{n}{\varvec{J}}_n\otimes \frac{1}{m}{{\varvec{M}}}^{-1}(\xi ) \\&+\frac{d}{1+\delta }\Big ({\varvec{I}}_n-\frac{1}{n}{\varvec{J}}_n\Big )\otimes ({{\varvec{e}}}_1{{\varvec{e}}}_1^T)\Big \}, \end{aligned}$$

where $\delta =md$ as defined in (13).

It follows that the lower bound in (10) is

$$\begin{aligned}&p + (n-1)\text {tr}\{({{\varvec{M}}}^{-1}(\xi ) + {\varvec{\varDelta }})^{-1}{\varvec{\varDelta }}\} \\&\quad = p+(n-1)\delta {{\varvec{e}}}_1^T({{\varvec{M}}}^{-1}(\xi ) +\delta {{\varvec{e}}}_1{{\varvec{e}}}_1^T )^{-1}{{\varvec{e}}}_1\\&\quad = p+(n-1)\delta {{\varvec{e}}}_1^T \left( {{\varvec{M}}}(\xi )-\displaystyle \frac{\delta {{\varvec{M}}}(\xi ){{\varvec{e}}}_1{{\varvec{e}}}_1^T{{\varvec{M}}}(\xi )}{1+\delta {{\varvec{e}}}_1^T{{\varvec{M}}}(\xi ){{\varvec{e}}}_1}\right) {{\varvec{e}}}_1\\&\quad = p+(n-1)\displaystyle \frac{\delta }{1+\delta }, \end{aligned}$$

which does not depend on $\xi$. If $\xi ^*$ is *D*-optimal for the fixed-effects model, we have

$$\begin{aligned} \max _{x\in {\mathcal {X}}}\phi (x,\xi ^*)=p+(n-1)\frac{\delta }{1+\delta },\end{aligned}$$

and it follows that the design $\xi ^*$ is *G*-optimal for prediction of individual parameters. Therefore, the *D*-optimal and *G*-optimal designs are equivalent for prediction in the random-intercept model.

## Conclusions

*G*-optimal designs are challenging to determine and study because the criterion is not differentiable and they require solving two or more layers of nested optimization problems over different spaces. However, the criterion is compelling and should appeal to researchers interested to design an experiment to estimate the overall response surface. To facilitate greater use of such designs for linear models with random effects, we proposed an effective meta-heuristic algorithm call CSO to find *G*-optimal designs and developed an equivalence theorem to confirm whether a design is *G*-optimal.

Additionally, we showed CSO is flexible and can also search for locally *D*-optimal designs for Poisson mixed regression models with several interacting factors, where some random effects may be correlated. We also provide R-codes freely and the interested reader can request them from the third author.

## Appendix

In this appendix, we show that the *G*-optimality criterion is a convex function and provide proofs for Theorems 1 and 2.

To show the *G*-optimality criterion is convex, we first let $\xi _{\alpha }=\alpha \xi +(1-\alpha ){\bar{\xi }}, 0<\alpha <1$, where $\xi$ and ${\bar{\xi }}$ are any two approximate designs in $\varXi$ and $\alpha \in [0,1]$. Then we have

$$\begin{aligned} {{\varvec{M}}}^{-1}(\xi _{\alpha })\le \alpha {{\varvec{M}}}^{-1}(\xi )+(1-\alpha )M^{-1}({\bar{\xi }}), \end{aligned}$$

and

$$\begin{aligned}&{\varvec{\varDelta }} - {\varvec{\varDelta }}({\varvec{M}}^{-1}(\xi _{\alpha })+{\varvec{\varDelta }})^{-1}{\varvec{\varDelta }} \\&\quad ={\varvec{M}}^{-1}(\xi _{\alpha })({\varvec{M}}^{-1}(\xi _{\alpha })+{\varvec{\varDelta }})^{-1}{\varvec{\varDelta }}\\&\quad =[({\varvec{M}}^{-1}(\xi _{\alpha })+{\varvec{\varDelta }}){\varvec{M}}(\xi _{\alpha })]^{-1}{\varvec{\varDelta }}\\&\quad =[I_p+{\varvec{\varDelta }}{\varvec{M}}(\xi _{\alpha })]^{-1}{\varvec{\varDelta }}\\&\quad =[\alpha (I_p+{\varvec{\varDelta }}{\varvec{M}}(\xi ))+(1-\alpha )(I_p+{\varvec{\varDelta }}{\varvec{M}}({\bar{\xi }}))]^{-1}{\varvec{\varDelta }}\\&\quad \le \alpha [I_p+{\varvec{\varDelta }}{\varvec{M}}(\xi ))]^{-1}{\varvec{\varDelta }}+(1-\alpha )[I_p+{\varvec{\varDelta }}{\varvec{M}}({\bar{\xi }})]^{-1}{\varvec{\varDelta }}. \end{aligned}$$

It follows that

$$\begin{aligned}&\phi (x,\xi _{\alpha })\\&\quad ={{\varvec{f}}}^\top (x)[{\varvec{M}}^{-1}(\xi _{\alpha })+(n-1)({\varvec{\varDelta }}- {\varvec{\varDelta }}({\varvec{M}}^{-1}(\xi _{\alpha })\\&\qquad +\,{\varvec{\varDelta }})^{-1}{\varvec{\varDelta }})]{{\varvec{f}}}(x)\\&\quad \le {{\varvec{f}}}^\top (x)[(\alpha {{\varvec{M}}}^{-1}(\xi )+(1-\alpha )M^{-1}({\bar{\xi }}))\\&\qquad +\,(n-1)(\alpha [I_p+{\varvec{\varDelta }}{\varvec{M}}(\xi )]^{-1}{\varvec{\varDelta }} \\&\qquad +\,(1-\alpha )[I_p+{\varvec{\varDelta }}{\varvec{M}}({\bar{\xi }})]^{-1}{\varvec{\varDelta }})]{{\varvec{f}}}(x)\\&\quad \le \alpha {{\varvec{f}}}^\top (x)({{\varvec{M}}}^{-1}(\xi )+(n-1)[I_p+{\varvec{\varDelta }}{\varvec{M}}(\xi )]^{-1}{\varvec{\varDelta }}){{\varvec{f}}}(x)\\&\qquad +\,(1-\alpha ){{\varvec{f}}}^\top (x)(M^{-1}({\bar{\xi }})\\&\qquad +\,(n-1)[I_p+{\varvec{\varDelta }}{\varvec{M}}({\bar{\xi }})]^{-1}{\varvec{\varDelta }}){{\varvec{f}}}(x)\\&\quad =\alpha \phi (x,\xi )+(1-\alpha )\phi (x,{\bar{\xi }}), \end{aligned}$$

which implies that

$$\begin{aligned} \psi _G^\mathrm{pred}(\xi )= & {} \max _{x\in {\mathcal {X}}}\phi (x,\xi _{\alpha })\\\le & {} \max _{x\in {\mathcal {X}}}(\alpha \phi (x,\xi )+(1-\alpha )\phi (x,{\bar{\xi }}))\\\le & {} \alpha \max _{x\in {\mathcal {X}}}\phi (x,\xi )+(1-\alpha )\max _{x\in {\mathcal {X}}}\phi (x,{\bar{\xi }})\\= & {} \alpha \psi _G^\mathrm{pred}(\xi )+(1-\alpha )\psi _G^\mathrm{pred}({\bar{\xi }}). \end{aligned}$$

This completes the argument that the *G*-optimality criterion is a convex functional.

### Proof of Theorem 1

For any design $\xi \in \varXi$, from (9), we have

$$\begin{aligned}&\int _{{\mathcal {X}}}\phi (x,\xi ) \mathrm{d}\xi \\&\quad =\int _{{\mathcal {X}}}\text {tr}[{{\varvec{f}}}^T(x){{\varvec{M}}}^{-1}(\xi ){{\varvec{f}}}(x) \\&\qquad +\, (n-1){{\varvec{f}}}^T(x)({\varvec{\varDelta }}- {\varvec{\varDelta }}({{\varvec{M}}}^{-1}(\xi )+{\varvec{\varDelta }})^{-1}{\varvec{\varDelta }}){{\varvec{f}}}(x)]\mathrm{d}\xi \\&\quad =\int _{{\mathcal {X}}}\text {tr}[{{\varvec{M}}}^{-1}(\xi ){{\varvec{f}}}(x){{\varvec{f}}}^T(x) \\&\qquad +\, (n-1)({\varvec{\varDelta }}- {\varvec{\varDelta }}({{\varvec{M}}}^{-1}(\xi )+{\varvec{\varDelta }})^{-1}{\varvec{\varDelta }}){{\varvec{f}}}(x){{\varvec{f}}}^T(x)]\mathrm{d}\xi \\&\quad =\text {tr}\{[{{\varvec{M}}}^{-1}(\xi ) \\&\qquad +\, (n-1)({\varvec{\varDelta }}- {\varvec{\varDelta }}({{\varvec{M}}}^{-1}(\xi )+{\varvec{\varDelta }})^{-1}{\varvec{\varDelta }})]\\&\quad \qquad \int _{{\mathcal {X}}}{{\varvec{f}}}(x){{\varvec{f}}}^T(x)\mathrm{d}\xi \}\\&\quad =\text {tr}\{[{{\varvec{M}}}^{-1}(\xi \\&\qquad +\, (n-1)({{\varvec{M}}}^{-1}(\xi )({{\varvec{M}}}^{-1}(\xi )+{\varvec{\varDelta }})^{-1}{\varvec{\varDelta }})]{{\varvec{M}}}(\xi )\}\\&\quad = p + (n-1)\text {tr}\{({{\varvec{M}}}^{-1}(\xi ) + {\varvec{\varDelta }})^{-1}{\varvec{\varDelta }}\}. \end{aligned}$$

Consequently,

$$\begin{aligned} \int _{{\mathcal {X}}}\phi (x,\xi ) \mathrm{d}\xi\le & {} \int _{{\mathcal {X}}}\max _{x\in {\mathcal {X}}}\phi (x,\xi ) \mathrm{d}\xi =\max _{x\in {\mathcal {X}}}\phi (x,\xi )\int _{{\mathcal {X}}} \mathrm{d}\xi \\= & {} \max _{x\in {\mathcal {X}}}\phi (x,\xi ), \end{aligned}$$

which proves Theorem 1. $\square$

### Proof of Theorem 2

For the *G*-optimal criterion in (9), let $\nabla \phi (x,\xi )$ be the $p\times p$ matrix with elements given by

$$\begin{aligned} \{\nabla \phi (x,\xi )\}_{ij}=\frac{\mathrm{d} \phi (x,\xi )}{\mathrm{d} {{\varvec{M}}}{(\xi )}_{ij}}, \quad i,j=1,2,\ldots ,p. \end{aligned}$$

A direct calculation shows that

$$\begin{aligned}&\{\nabla \phi (x,\xi )\}_{ij}\\&\quad ={{\varvec{f}}}^\top (x)\frac{\mathrm{d}{{\varvec{M}}}^{-1}(\xi )}{\mathrm{d}{{\varvec{M}}}(\xi )_{ij}}{{\varvec{f}}}(x) \\&\qquad +\,(n-1){{\varvec{f}}}^\top (x)\frac{d [{\varvec{\varDelta }} - {\varvec{\varDelta }}({\varvec{M}}^{-1}(\xi )+{\varvec{\varDelta }})^{-1}{\varvec{\varDelta }}]}{\mathrm{d}{{\varvec{M}}}(\xi )_{ij}}{{\varvec{f}}}(x)\\&\quad =-\,{{\varvec{f}}}^\top (x){{\varvec{M}}}^{-1}(\xi )\frac{\mathrm{d}{{\varvec{M}}}(\xi )}{\mathrm{d}{{\varvec{M}}}(\xi )_{ij}}{{\varvec{M}}}^{-1}(\xi ){{\varvec{f}}}(x)\\&\qquad -\,(n-1){{\varvec{f}}}^\top (x){\varvec{\varDelta }}\frac{d [({\varvec{M}}^{-1}(\xi )+{\varvec{\varDelta }})^{-1}]}{\mathrm{d}{{\varvec{M}}}(\xi )_{ij}}{\varvec{\varDelta }}{{\varvec{f}}}(x)\\&\quad =-\,{{\varvec{f}}}^\top (x){{\varvec{M}}}^{-1}(\xi )E_{ij}{{\varvec{M}}}^{-1}(\xi ){{\varvec{f}}}(x)\\&\qquad +\,(n-1){{\varvec{f}}}^\top (x){\varvec{\varDelta }}({\varvec{M}}^{-1}(\xi )+{\varvec{\varDelta }})^{-1} \\&\qquad \times \frac{d [{\varvec{M}}^{-1}(\xi )+{\varvec{\varDelta }}]}{\mathrm{d}{{\varvec{M}}}(\xi )_{ij}}({\varvec{M}}^{-1}(\xi )+{\varvec{\varDelta }})^{-1}{\varvec{\varDelta }}{{\varvec{f}}}(x)\\&\quad =-\,{{\varvec{f}}}^\top (x){{\varvec{M}}}^{-1}(\xi )E_{ij}{{\varvec{M}}}^{-1}(\xi ){{\varvec{f}}}(x)\\&\qquad -\,(n-1){{\varvec{f}}}^\top (x){\varvec{\varDelta }}({\varvec{M}}^{-1}(\xi )+{\varvec{\varDelta }})^{-1}{\varvec{M}}^{-1}(\xi )\\&\qquad \times \frac{\mathrm{d}{\varvec{M}}(\xi )}{\mathrm{d}{{\varvec{M}}}(\xi )_{ij}}{\varvec{M}}^{-1}(\xi )({\varvec{M}}^{-1}(\xi )+{\varvec{\varDelta }})^{-1}{\varvec{\varDelta }}{{\varvec{f}}}(x)\\&\quad =-\,{{\varvec{f}}}^\top (x){{\varvec{M}}}^{-1}(\xi )E_{ij}{{\varvec{M}}}^{-1}(\xi ){{\varvec{f}}}(x)\\&\qquad -\,(n-1){{\varvec{f}}}^\top (x){\varvec{\varDelta }}({\varvec{M}}^{-1}(\xi )+{\varvec{\varDelta }})^{-1}{\varvec{M}}^{-1}(\xi )\\&\qquad \times E_{ij}{\varvec{M}}^{-1}(\xi )({\varvec{M}}^{-1}(\xi )+{\varvec{\varDelta }})^{-1}{\varvec{\varDelta }}{{\varvec{f}}}(x)\\&\quad =-\,{{\varvec{f}}}^\top (x){{\varvec{M}}}^{-1}(\xi )E_{ij}{{\varvec{M}}}^{-1}(\xi ){{\varvec{f}}}(x)\\&\qquad -\,(n-1){{\varvec{f}}}^\top (x)[{\varvec{\varDelta }} - {\varvec{\varDelta }}({\varvec{M}}^{-1}(\xi )+{\varvec{\varDelta }})^{-1}{\varvec{\varDelta }}]{\varvec{M}}^{-1}(\xi )\\&\qquad \times E_{ij}{\varvec{M}}^{-1}(\xi )[{\varvec{\varDelta }} - {\varvec{\varDelta }}({\varvec{M}}^{-1}(\xi )+{\varvec{\varDelta }})^{-1}{\varvec{\varDelta }}]{{\varvec{f}}}(x)\\&\quad =-\,{{\varvec{f}}}^\top (x){{\varvec{M}}}^{-1}(\xi )E_{ij}{{\varvec{M}}}^{-1}(\xi ){{\varvec{f}}}(x) \\&\qquad -\,(n-1){{\varvec{f}}}^\top (x)N(\xi ,{\varvec{\varDelta }})E_{ij}N(\xi ,{\varvec{\varDelta }}){{\varvec{f}}}(x)\\&\quad =-\,{{\varvec{f}}}^\top (x){{\varvec{M}}}^{-1}(\xi ){{\varvec{e}}}_i{{\varvec{e}}}_j^\top {{\varvec{M}}}^{-1}(\xi ){{\varvec{f}}}(x)\\&\qquad -\,(n-1){{\varvec{f}}}^\top (x)N(\xi ,{\varvec{\varDelta }}){{\varvec{e}}}_i{{\varvec{e}}}_j^\top N(\xi ,{\varvec{\varDelta }}){{\varvec{f}}}(x)\\&\quad =-\,{{\varvec{e}}}_j^\top {{\varvec{M}}}^{-1}(\xi ){{\varvec{f}}}(x){{\varvec{f}}}^\top (x){{\varvec{M}}}^{-1}(\xi ){{\varvec{e}}}_i \\&\qquad -\,(n-1){{\varvec{e}}}_j^\top N(\xi ,{\varvec{\varDelta }}){{\varvec{f}}}(x){{\varvec{f}}}^\top (x)N(\xi ,{\varvec{\varDelta }}){{\varvec{e}}}_i\\&\quad =-\,\{{{\varvec{M}}}^{-1}(\xi ){{\varvec{f}}}(x){{\varvec{f}}}^\top (x){{\varvec{M}}}^{-1}(\xi )\\&\qquad +\,(n-1)N(\xi ,{\varvec{\varDelta }}){{\varvec{f}}}(x){{\varvec{f}}}^\top (x)N(\xi ,{\varvec{\varDelta }})\}_{ji}, \end{aligned}$$

where $E_{ij}$ is the matrix with 1 in the (*i*, *j*)*th* position and zeros elsewhere and ${{\varvec{e}}}_j$ is the *j* *th* standard basis for $R^p$. It follows that

$$\begin{aligned} \nabla \phi (x,\xi )= & {} -{{\varvec{M}}}^{-1}(\xi ){{\varvec{f}}}(x){{\varvec{f}}}^\top (x){{\varvec{M}}}^{-1}(\xi )\\&-\,(n-1)N(\xi ,{\varvec{\varDelta }}){{\varvec{f}}}(x){{\varvec{f}}}^\top (x)N(\xi ,{\varvec{\varDelta }}), \end{aligned}$$

and

$$\begin{aligned}&\text {tr}\{{{\varvec{f}}}(x){{\varvec{f}}}^\top (x)\int _{{\mathcal {A}}(\xi )}\nabla \phi (z,\xi )\mathrm{d}\mu \}\\&\quad =\text {tr}\{{{\varvec{f}}}(x){{\varvec{f}}}^\top (x)[-{{\varvec{M}}}^{-1}(\xi ){{\varvec{M}}}_{{\mathcal {A}}}(\mu ){{\varvec{M}}}^{-1}(\xi )\\&\qquad -\,(n-1)N(\xi ,{\varvec{\varDelta }}){{\varvec{M}}}_{{\mathcal {A}}}(\mu )N(\xi ,{\varvec{\varDelta }})]\}\\&\quad =-\,{{\varvec{f}}}^\top (x){{\varvec{M}}}^{-1}(\xi ){{\varvec{M}}}_{{\mathcal {A}}}(\mu ){{\varvec{M}}}^{-1}(\xi ){{\varvec{f}}}(x)\\&\qquad -\,(n-1){{\varvec{f}}}^\top (x)N(\xi ,{\varvec{\varDelta }}){{\varvec{M}}}_{{\mathcal {A}}}(\mu )N(\xi ,{\varvec{\varDelta }}){{\varvec{f}}}(x),\\&\qquad \quad \text {tr}\{{{\varvec{M}}}(\xi )\int _{{\mathcal {A}}(\xi )}\nabla \phi (z,\xi )\mathrm{d}\mu \}\\&\quad =-\text {tr}\{{{\varvec{M}}}_{{\mathcal {A}}}(\mu ){{\varvec{M}}}^{-1}(\xi )+(n-1){{\varvec{M}}}(\xi )N(\xi ,{\varvec{\varDelta }})\\&\qquad \quad {{\varvec{M}}}_{{\mathcal {A}}}(\mu )N(\xi ,{\varvec{\varDelta }})\}. \end{aligned}$$

$$\square $$

## References

[CR1] Berger M, Wong WK (2005). Applied optimal designs.

[CR2] Berger M, Wong WK (2009). An introduction to optimal designs with applications to social and biomedical research.

[CR3] Berger M, King CY, Wong WK (2000). Minimax $${D}$$-optimal designs for item response theory models. Psychometrika.

[CR4] Chen RB, Chen PY, Wong WK (2018). Standardized maximin $${D}$$-optimal designs for pharmacological models via particle swarm optimization techniques. Chemom Intell Lab Syst.

[CR5] Cheng R, Jin Y (2014). A competitive swarm optimizer for large scale optimization. IEEE Trans Cybern.

[CR6] Debusho LK, Haines LM (2008). $${V}$$ and $${D}$$-optimal population designs for the simple linear regression model with a random intercept term. J Stat Plan Inference.

[CR7] Debusho LK, Haines LM (2011). $${D}$$ and $${V}$$-optimal population designs for the quadratic regression model with a random intercept term. J Stat Plan Inference.

[CR8] Entholzner M, Benda N, Schmelter T, Schwabe R (2005). A note on designs for estimating population parameters. Biom Lett Listy Biom.

[CR9] Fedorov V (1972). Optimal theory of experimental designs.

[CR10] Fedorov V, Hackl P (1972). Model-oriented design of experiments.

[CR11] Gu S, Cheng R, Jin Y (2018). Feature selection for high-dimensional classification using a competitive swarm optimizer. Soft Comput.

[CR12] Huang MNL, Chang F-C, Wong WK (1995). D-optimal designs for polynomial regression without an intercept. Stat Sin.

[CR13] Kiefer J (1959). Optimum experimental designs. J R Stat Soc Ser B (Methodol).

[CR14] Kiefer J, Wolfowitz J (1960). The equivalence of two extremum problems. Can J Math.

[CR15] Kim S, Wong WK (2018). Extended two-stage adaptive designs with three target responses for phase ii clinical trials. Stat Methods Med Res.

[CR16] Kumarappan N, Arulraj R (2016) Optimal installation of multiple dg units using competitive swarm optimizer (cso) algorithm. In: 2016 IEEE Congress on evolutionary computation (CEC). IEEE, pp 3955–3960

[CR17] Masoudi E, Holling H, Duarte BP, Wong WK (2019). A metaheuristic adaptive cubature based algorithm to find bayesian optimal designs for nonlinear models. J Comput Graph Stat.

[CR18] Mohapatra P, Das KN, Roy S (2017). A modified competitive swarm optimizer for large scale optimization problems. Appl Soft Comput.

[CR19] Naderi D, Niaparast M, Zangenehmehr A (2018). D-optimal designs for multiple poisson regression model with random coefficients. Asian Res J Math.

[CR20] Niaparast M, Schwabe R (2013). Optimal design for quasi-likelihood estimation in poisson regression with random coefficients. J Stat Plan Inference.

[CR21] Pazman A (1986). Foundations of optimum experimental designs.

[CR22] Phoa FKH, Chen R-B, Wang W, Wong WK (2016). Optimizing two-level supersaturated designs using swarm intelligence techniques. Technometrics.

[CR23] Prus M (2019). Various optimality criteria for the prediction of individual response curves. Stat Probab Lett.

[CR24] Prus M, Schwabe R (2016a) Interpolation and extrapolation in random coefficient regression models: optimal design for prediction. In: mODa 11-advances in model-oriented design and analysis. Springer, pp 209–216

[CR25] Prus M, Schwabe R (2016b) Optimal designs for the prediction of individual parameters in hierarchical models. J R Stat Soc Ser B (Stat Methodol) 78(1):175–191

[CR26] Royston P, Altman DG (1994). Regression using fractional polynomials of continuous covariates: parsimonious parametric modelling. J R Stat Soc Ser C (Appl Stat).

[CR27] Royston P, Sauerbrei W (2008). Multivariable model-building: a pragmatic approach to regression analysis based on fractional polynomials for modelling continuous variables.

[CR28] Royston P, Ambler G, Sauerbrei W (1999). The use of fractional polynomials to model continuous risk variables in epidemiology. Int J Epidemiol.

[CR29] Schmelter T (2007a) Considerations on group-wise identical designs for linear mixed models. J Stat Plan Inference 137(12):4003–4010

[CR30] Schmelter T (2007b) The optimality of single-group designs for certain mixed models. Metrika 65(2):183–193

[CR31] Storn R, Price K (1997). Differential evolution-a simple and efficient heuristic for global optimization over continuous spaces. J Glob Optim.

[CR32] Sun C, Ding J, Zeng J, Jin Y (2018). A fitness approximation assisted competitive swarm optimizer for large scale expensive optimization problems. Memet Comput.

[CR33] Walters GD (2007a) Predicting institutional adjustment with the lifestyle criminality screening form and the antisocial features and aggression scales of the pai. J Pers Assess 88(1):99–105 10.1080/00223890709336840 17266420

[CR34] Walters GD (2007b) Using $${P}$$oisson class regression to analyze count data in correctional and forensic psychology: a relatively old solution to a relatively new problem. Crim Justice Behav 34(12):1659–1674

[CR35] Whitacre JM (2011a) Recent trends indicate rapid growth of nature-inspired optimization in academia and industry. Computing 93(2–4):121–133

[CR36] Whitacre JM (2011b) Survival of the flexible: explaining the recent popularity of nature-inspired optimization within a rapidly evolving world. Computing 93(2–4):135–146

[CR37] Wong WK, Cook RD (1993). Heteroscedastic G-optimal designs. J R Stat Soc Ser B (Methodol).

[CR38] Xiong G, Shi D (2018). Orthogonal learning competitive swarm optimizer for economic dispatch problems. Appl Soft Comput.

[CR39] Yang XS (2010). Nature-inspired metaheuristic algorithms.

[CR40] Zhang WX, Chen WN, Zhang J (2016) A dynamic competitive swarm optimizer based-on entropy for large scale optimization. In: 2016 eighth international conference on advanced computational intelligence (ICACI). IEEE, pp 365–371

[CR41] Zhang Z, Wong WK, Tan KC (2020) Competitive swarm optimizer with mutated agents for finding optimal designs for nonlinear regression models with multiple interacting factors. Memet Comput 12(3): 219–233 10.1007/s12293-020-00305-6 PMC7968042 33747240

[CR42] Zhou J, Fang W, Wu X, Sun J, Cheng S (2016) An opposition-based learning competitive particle swarm optimizer. In: 2016 IEEE Congress on evolutionary computation (CEC). IEEE, pp 515–521

